# Src and β-catenin control a senescence-fibrotic phenotype in uterine fibroid cells

**DOI:** 10.1038/s41598-026-57742-5

**Published:** 2026-06-26

**Authors:** Md Soriful Islam, Valeria I. Macri, Lauryn Martin, Claire Shen, Joscelyn Mejias, Samya El Sayed, Rachel Michel, Casie Post, Bhuchitra Singh, Joshua T. Brennan, Abigail Fennell, Jude M. Phillip, Mostafa A. Borahay, Jennifer H. Elisseeff, James H. Segars

**Affiliations:** 1 Division of Reproductive Sciences and Women’s Health Research, Department of Gynecology and Obstetrics, Johns Hopkins Medicine, 720 Rutland Ave, Ross Building, Room 624, Baltimore, MD 21205 USA; 2 Translational Tissue Engineering Center, Johns Hopkins University School of Medicine, Baltimore, MD 21205 USA; 3 Department of Biomedical Engineering, Johns Hopkins University, Baltimore, MD 21212 USA; 4 Institute for Nanobiotechnology, Johns Hopkins University, Baltimore, MD 21212 USA; 5 Department of Chemical and Biomolecular Engineering, Johns Hopkins University, Baltimore, MD 21212 USA; 6 Department of Oncology, Sidney Kimmel Comprehensive Cancer Center, Johns Hopkins School of Medicine, Baltimore, MD 21287 USA

**Keywords:** Uterine fibroids, Substrate stiffness, Senescence, Fibrosis, β-Catenin, Senolytics, Dasatinib, Cell biology, Diseases

## Abstract

Uterine fibroids (or leiomyomas) are highly prevalent benign tumors in women of reproductive age and are associated with abnormal uterine bleeding, pelvic pain, and infertility. Fibroid tumors are characterized by excessive extracellular matrix deposition and increased tissue stiffness, however the functional impact of this mechanical property in regulating fibroid cell behavior remains poorly understood. Uterine fibroids feature and increased number of senescent cells accompanied by profibrotic and inflammatory signaling but their contribution to fibroid growth is not fully understood. We observed that mechanical stiffness promoted senescence and fibrotic phenotypes in uterine fibroid cells. Fibroid cells cultured on stiff (⁓ 3 GPa) compared to soft (930 kPa) substrates exhibited elevated expression of senescence markers, including p16 and p21, as well as increased levels of fibrosis associated proteins such as fibronectin, PAI1, and α-SMA. Mechanistically, we identified Src and β-catenin as key regulators of this phenotype. Furthermore, the senolytic Src inhibitor dasatinib reduced senescence and fibrosis markers and attenuated β-catenin activity, suggesting that Src acts upstream of β-catenin in this context. Inhibition of β-catenin using ICG001 or PRI724 suppressed senescence and fibrosis markers and reduced downstream targets including CTGF and cyclin D1. These findings reveal a mechanistic link between mechanical stiffness, cellular senescence, and fibrosis in uterine fibroids, and identify Src and β-catenin as potential therapeutic targets.

**Supplementary Information:**

The online version contains supplementary material available at https://doi.org/10.1038/s41598-026-57742-5.

## Introduction

Uterine fibroids (or leiomyomas) are the most common benign tumors in women of reproductive age with a prevalence exceeding 70% in White women and 80% in African American women by the age of 50^1^. Fibroid tumors are associated with debilitating symptoms, including heavy menstrual bleeding, pelvic pain, and infertility, leading to a significant impact on the quality of life^2,3^. The economic burden of uterine fibroids in the United States was estimated to be $42.2 billion in 2022^4,5^. Despite their high prevalence and clinical and economic impact, the molecular mechanisms underlying fibroid pathogenesis remain poorly understood, hindering the development of effective non-invasive therapies.

A hallmark of uterine fibroids is the accumulation of extracellular matrix (ECM) components, including collagens and fibronectin, which contribute to elevated tissue stiffness^6–10^. Increased expression of mechanoresponsive transcriptional regulators, such as YAP and β-catenin, has been observed in fibroid tissues, reflecting the stiff microenvironment of the tumors^6,11,12^. Mechanical rigidity of the tissue microenvironment has been shown to promote cellular senescence and fibrosis in various fibrotic and tumor contexts^13,14^. However, the functional impact of mechanical stiffness to regulate senescence and fibrosis in fibroid cells remains poorly understood.

Cellular senescence, characterized by irreversible cell cycle arrest, and a pro-inflammatory secretory phenotype known as the senescence-associated secretory phenotype (SASP), plays a critical role in aging and a wide range of pathological conditions, including fibrosis, by sustaining chronic inflammation and ECM remodeling^15,16^. Ongoing clinical trials are evaluating the senolytic agents dasatinib and quercetin for their ability to selectively eliminate senescent cells in human disease^17^. Accumulating evidence suggests that senescent cells are present in fibroid tumors^18–20^; however, their functional contribution to fibroid growth remains incompletely understood. Collectively, these observations led us to probe the mechanistic link between mechanical stiffness, cellular senescence, and fibrosis in uterine fibroids.

To study the effects of mechanical stiffness, we used silicone (930 kPa) and polystyrene (~ 3 GPa) substrates to model soft and stiff microenvironments^21^. We assessed the expression of senescence markers (p16 and p21), fibrosis-associated proteins (fibronectin, PAI-1, and α-SMA)^22–24^, and mechanotransduction pathways including β-catenin^11^. In addition, the senolytic compound dasatinib (a Src inhibitor) was evaluated for its ability to modulate senescence and fibrotic responses. Src activity has been previously reported in uterine fibroids, with detectable phosphorylated Src in fibroid cells and functional evidence supporting its role in leiomyoma growth^25–27^; however, its specific contribution to senescence- and fibrosis-associated phenotypes remains poorly understood. To determine the role of β-catenin in regulating senescence and fibrotic phenotypes, we used two pharmacological inhibitors, ICG-001 and PRI-724. Our findings revealed that mechanical stiffness promoted the expression of senescence and fibrosis-associated markers in fibroid cells. The pharmacological inhibition of Src and β-catenin signaling reduced these markers, identifying these pathways as key regulators of the senescence-fibrotic phenotype in fibroid cells.

## Materials and methods

### Materials

Soft (930 kPa) silicone plates (cat. no. UF-4001C for 6-well and cat no. TPW-3001 for 24-well) were obtained from Flexcell International Corporation, and stiff (~ 3 GPa) polystyrene plates (cat. no. 354400 for 6-well and cat no. 354408 for 24-well) were sourced from Corning. Dasatinib (cat. no. HY-10181) was obtained from MedChemExpress and ICG-001 (cat. no. S8968) and PRI-724 or Foscenvivint (cat. no. S8968) were purchased from Selleck Chemicals. All the chemicals were dissolved in DMSO and stored at either − 20 °C or − 80 °C, as per manufacturer instructions.

### Primary human uterine fibroid cell culture

Fibroid and myometrial tissue samples were obtained from premenopausal women who underwent elective hysterectomy. The research was approved by the institutional review board of Johns Hopkins University. Written informed consent was obtained from all participants. All research was performed in accordance with relevant US Federal guidelines/regulations and the study is registered on the NIH Clinical Trials database (NCT06135870). All human subjects research was conducted in accordance with the Declaration of Helsinki. Fibroid samples were procured from women aged 38 to 53 years, representing both Black/African American and White ethnic groups. Fibroid sizes ranged from 1.5 to 12 cm in diameter and were identified in intramural, submucosal, or subserosal regions of the uterus, based on established histopathological criteria. None of the participants used GnRH analogues or anti-progestins within three months prior to surgery. Upon surgical removal, the fibroid tissues were immediately placed in Hanks’ Balanced Salt Solution (HBSS; Gibco, cat no. 14025-092) containing gentamicin (Sigma, cat no. G1397) and normocin (InvivoGen, cat no. ant-nr-1) and transported to the laboratory. The tissues were finely chopped and incubated in a solution of HBSS supplemented with 1.5 mg/ml collagenase type IV (Gibco, cat no. 17104-019) or collagenase type I (Sigma, cat no. C0130-1G), DNase (Sigma, cat no. D5025-150KU), CaCl_2_ (Quality Biological, cat no. 351-130-721), and HEPES (Gibco, cat no. 15630-080). The incubation was carried out at 37 °C with constant agitation for 4 to 8 h. Following enzymatic digestion, the cell suspension was sequentially filtered through 100 µm (CELLTREAT, cat no. 229485) strainers. The filtered suspension was centrifuged twice at 1500 rpm for 10 min, and the supernatant was discarded. The cell pellet was resuspended in T75 or T25 culture flasks containing DMEM-F12 medium (Gibco, cat no. 11330-032) supplemented with 10% fetal bovine serum (FBS; Cytiva, SH30910.03) and 100 µg/L normocin. Cultures were maintained in a 37 °C incubator with 5% CO_2_. After 48 to 72 h, non-adherent or dead cells were removed by washing with phosphate-buffered saline (PBS; Gibco, cat no. 10010–023), followed by the addition of cell culture media. Primary cell cultures of low passage numbers (1–5) were used to assess cell responses.

### Cell culture and treatments

To investigate the effect of substrate stiffness on senescence- and fibrosis-associated proteins, primary uterine fibroid cells were cultured on substrates of defined stiffness: soft silicone (930 kPa) (Flexcell, cat. no. UF-4001C for 6-well plates and cat no. TPW-3001 for 24-well plates) and stiff polystyrene (~ 3 GPa) (Corning, Corning, cat. no. 354400 for 6-well plates and cat no. 354408 for 24-well plates), as previously described^21^. Cells were cultured for 24 or 48 h for mRNA quantification by quantitative real-time PCR^28,29^, for 48 h for protein quantification by western blotting^28,30^, or for 5 days for visualization of p16 and p21 protein expression by immunofluorescence^31^. Shorter time points (24 h) for RNA measurement were used to assess early transcriptional regulation, while longer time points (48 h) were used when evaluating downstream or sustained gene expression changes following pharmacological inhibition. To evaluate the impact of Src inhibition and senolytic potential, fibroid cells cultured on stiff substrates were treated with dasatinib at concentrations of 0.02, 0.1, 0.5, or 1 µM for 48 h, or with vehicle control (DMSO). Control cells received the same final concentration of DMSO (0.01%) as that used in the highest dasatinib treatment. To assess the role of β-catenin signaling in the regulation of senescence and fibrotic phenotypes, fibroid cells cultured on stiff substrates were treated with the β-catenin inhibitors ICG-001 (2.5 µM for 48 h) or PRI-724 (0.3–1.25 µM for 48 h), followed by analysis of gene and protein expression. Control cells received the same final concentration of DMSO (0.05% for ICG-001 and 0.01% for PRI-724) as used in the corresponding highest drug concentrations. For cell viability assays, fibroid cells cultured on stiff substrates were treated with dasatinib (0.02–2 µM), ICG-001 (0.5–20 µM) or PRI-724 (0.156–2.5 µM) for 24 or 48 h, and viability was assessed using the CellTiter 96® AQueous One Solution Cell Proliferation Assay (MTS; Promega, cat no. G3588). Control cells received the same final concentration of DMSO (0.02% for dasatinib, 0.04% for ICG-001, and 0.025% for PRI-724) as used in the corresponding highest drug concentrations.

### Western blot

Protein lysates were prepared by lysing cells in RIPA buffer (Sigma, cat no. R0278) supplemented with a protease and phosphatase inhibitor cocktail (Cell Signaling Technology, cat no. 5872S) after rinsing with PBS. The protein concentrations were quantified using the Pierce™ BCA Protein Assay Kit (ThermoFisher Scientific, cat no. 23227). For electrophoresis, 30–50 µg of protein was loaded onto 4–12% NuPAGE gels (Invitrogen, cat no. NP0321BOX or NP0322BOX) and separated under reducing conditions by SDS-PAGE. PageRuler™ Plus Prestained Protein Ladder (Thermo Fisher Scientific, cat no. 26619) was used a molecular size standard (Supplementary File 1, Fig. S1). The resolved proteins were transferred to 0.2-µm nitrocellulose membranes (Novex, cat no. LC2000) using either the XCell SureLock system (Novex, cat no. EI0001) or the iBlot 3 Western Blot Transfer System (Invitrogen, cat no. IB31001). Ponceau S solution (Sigma, cat no. P7170) was applied to confirm the efficiency of protein transfer. Membranes were then segmented based on the molecular weights of the target proteins and blocked for 20 min in 5% non-fat milk (BIO-RAD, cat. no. 1706404) diluted in TBST (1X Tris-Buffered Saline with 0.1% Tween 20). Subsequently, membranes were incubated with primary antibodies specific to p16, p21, fibronectin, plasminogen activator inhibitor-1 (PAI-1), α-Smooth muscle actin (α-SMA), active β-catenin, non-phosphorylated YAP, phospho-Smad2, cyclin D1, connective tissue growth factor (CTGF), SOX9, COL1A1, phospho-Src, LAMC1, MMP2, TIMP1, and α-actinin (Table S1) either overnight at 4 °C or for one hour at room temperature. Following three washes with TBST (5 min each), membranes were incubated with horseradish peroxidase (HRP)-conjugated secondary antibodies (1:5000 dilution; GE Healthcare, cat no. NA934V or NA931V) in 5% non-fat milk with TBST for 2 h at room temperature. After another round of three 5-min washes, immunoreactive bands were visualized using either the SuperSignal™ West Pico PLUS Chemiluminescent Substrate (ThermoFisher Scientific, cat no. 34580) or the SuperSignal™ West Atto Ultimate Sensitivity Chemiluminescent Substrate (ThermoFisher Scientific, cat. no. A38556) and imaged with an Azure Imager c300 system (Azure Biosystems). Band intensities were analyzed with Image J 1.52a software, normalized against the loading control α-actinin.

### Quantitative real time PCR

RNA extraction and purification were carried out using RLT buffer, followed by processing with the RNeasy Mini Kit (Qiagen, 74106). The quality and concentration of RNA were measured with a NanoVue Plus spectrophotometer (Biochrom US). Complementary DNA (cDNA) was synthesized from 50 to 100 ng/µL of RNA using the iScript™ cDNA Synthesis Kit (Bio-Rad, cat. No. 1708891) in a Bio-Rad Thermocycler. Quantitative real-time PCR (qRT-PCR) was performed on a LightCycler® 96 System (Roche Diagnostics) in 96-well plates. The reaction mixture consisted of 1.6 ng/µL cDNA, 1X FastStart Essential DNA Green Master (Roche Diagnostics, cat no. 06402712001), and gene-specific primer sets (Table S2) in a total volume of 10 µL. The forward and reverse primers (synthesized by IDT) were designed to evaluate mRNA expression levels of various genes, including *CDKN2A, CDKN1A, FN1, PAI1, ACTA2, VCAN, TNC, LAMC1, POSTN, COL1A1, COL3A1, COL4A2, COL5A1, COL6A1, LOX1, P4HA1, P4HA2, GLB1, TGFB1, IGF1,* and *ARF1* (Table S2). *ARF1* was used as a reference gene for normalization of qRT-PCR data. Relative mRNA expression levels were determined using the ΔΔCt method and are expressed as fold changes compared to control samples.

### Immunofluorescence

Cells were cultured in 24-well plates coated with collagen (soft plate: Flexcell, cat. no. TPW-3001; stiff plate: Corning, cat. no. 354408) or uncoated (stiff plate: Corning, cat. no. 353047). Cells were fixed with 4% formaldehyde in 1 × PBS at room temperature for 12–15 min, followed by three consecutive washes with 1 × PBS, each lasting 5 min. To minimize nonspecific binding, cells were incubated for 1 h in a blocking solution composed of 1X PBS, 5% Normal Goat Serum, and 0.3% Triton X-100. After blocking, cells were exposed to the primary antibodies p16, p21, CTGF, COL1A1, fibronectin (Table S1) overnight at 4 °C or for 1 h at room temperature in an antibody dilution buffer containing 1X PBS, 1% BSA, and 0.3% Triton X-100. The next day, the cells were washed three times with 1X PBS for 5 min per wash and then incubated in the dark for 2 h at room temperature with the secondary antibody Alexa Fluor™ Plus 488 goat anti-rabbit IgG (H + L) (2 mg/ml) (Invitrogen, A32731) diluted in the same antibody dilution buffer. Following secondary antibody incubation, cells were three additional 5-min washes with 1X PBS before being mounted with ProLong Gold antifade reagent containing DAPI (Cell Signaling Technology, 8961). The slides were left to dry overnight at room temperature in a dark environment before storage at 4 °C. Images were obtained at 10X magnification using the Echo Revolve microscope system (Echo).

### Statistical analysis

All statistical analyses and data visualization were performed using GraphPad Prism 10. The specific statistical tests applied are described in the corresponding figure legends. The Shapiro–Wilk test was used to assess the normality of the data. For comparisons, an unpaired t-test, one-way ANOVA, or Kruskal–Wallis test was performed as appropriate. P-value of less than 0.05 was considered statistically significant.

## Results

### A stiff substrate was associated with increased expression of senescence markers in uterine fibroid cells

**Fig. 1:**
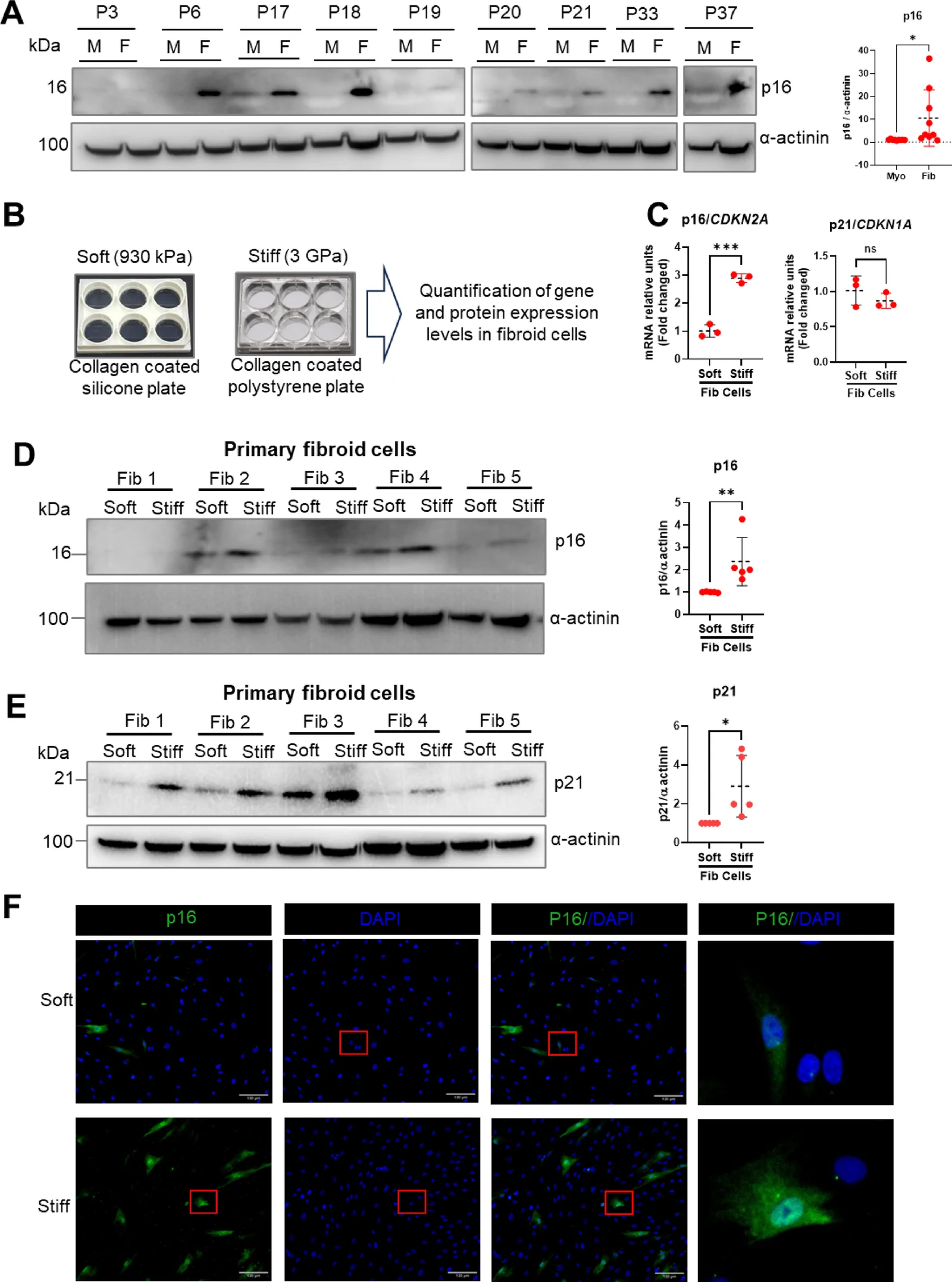
Substrate stiffness increased expression of senescence markers in cultured uterine fibroid cells. (**A**) Western blot images (left panel) and quantification (right panel) of p16 in primary myometrial and fibroid cells isolated from patient-matched tissues (myo = 9, fib = 9 from 9 patients) presented in scatter plots. (**B**) Schematic diagram of experimental design for quantification of gene and protein expression levels from cultured fibroid cells cultured on a soft silicone (930 kPa) and a stiff polystyrene substrate (3 GPa) for 24–48 h or 5 days. (**C**) Scatter plots showing the mRNA levels of p16 (*CDKN2A*) and p21 (*CDKN1A*) in primary fibroid cells cultured on soft and stiff substrates for 24 h (soft = 3, stiff = 3 replicates from independent cultures). *ARF1* was used as a housekeeping gene to normalize the gene expression data. (**D**, **E**) Western blot images (left panel) and quantification (right panel) of p16 and p21 in primary fibroid cells cultured on a soft (930 kPa) and a stiff substrate (3 GPa) for 48 h (soft = 5, stiff = 5 5 fibroids from 5 patients) presented in scatter plots. Full immunoblots related to Fig. 1 are shown in Supplementary File 2, Fig. S1. Membranes were cut into several pieces (based on the molecular weight of proteins of interest) prior to hybridization with primary antibodies (p16, p21, and α-actinin) during western blot. MW (in kDa) are indicated. The α-actinin was used as a loading control to normalize the protein expression data. (**F**) Representative 10X immunofluorescence images of p16 from four biological replicates of primary fibroid cells cultured on soft and stiff substrates for 5 days. Shapiro–Wilk test was used to assess whether data were normally distributed. *p < 0.05; **p < 0.01; ***p < 0.001. Unpaired t test (**A, C**–**E**).

Previous studies have demonstrated the presence of senescent cells in uterine fibroids^20,32,33^; however, the factors responsible for inducing senescence in fibroid cell populations remain largely undefined. The tumor microenvironment, particularly mechanical stiffness, plays a critical role in the pathogenesis of various tumors^34^. Mechanical stiffness is a hallmark of uterine fibroids^8,10,35–37^, but its specific contribution to the induction of senescence in fibroid cells has not been studied. To address this, we first confirmed the presence of senescent cells in fibroid tissues by comparing senescence marker expression levels in paired myometrial and fibroid cells. Myometrial and fibroid cells were isolated from fresh tissue samples by enzymatic digestion overnight with collagenase type I, followed by sequential filtration and centrifugation. Protein lysates were prepared using RIPA buffer and analyzed by western blot. We found that p16 protein levels were markedly elevated in fibroid cells, showing a 10.5-fold increase compared to myometrial cells (Fig. 1A), confirming an enrichment of senescent cells in fibroid populations. We next examined whether substrate stiffness contributed to the induction of senescence in fibroid cells. Primary fibroid cells were cultured for 48 h on soft (930 kPa) or stiff (~ 3 GPa) substrates^21^, and senescence marker expression was quantified. P16 (*CDKN2A*) mRNA expression was 2.9-fold higher in cells cultured on the stiff compared to soft substrate (Fig. 1C) and was accompanied by a parallel 2.5-fold increase in p16 protein levels (Fig. 1D). Although p21 (*CDKN1A*) mRNA levels did not differ significantly between conditions (Fig. 1C), p21 protein expression was significantly elevated in cells cultured on the stiff substrate (Fig. 1E). These findings were further supported by immunofluorescence staining, with increased p16 and p21 protein levels under stiff culture conditions (Fig. 1F and Supplementary File 1, Fig. S2). Overall, these data indicate that senescent cells are a component of fibroid cell populations and that mechanical stiffness may contribute to the induction of the senescence phenotype in uterine fibroid cells.

### A stiff substrate altered expression of fibrosis-associated proteins in cultured uterine fibroid cells

**Fig. 2:**
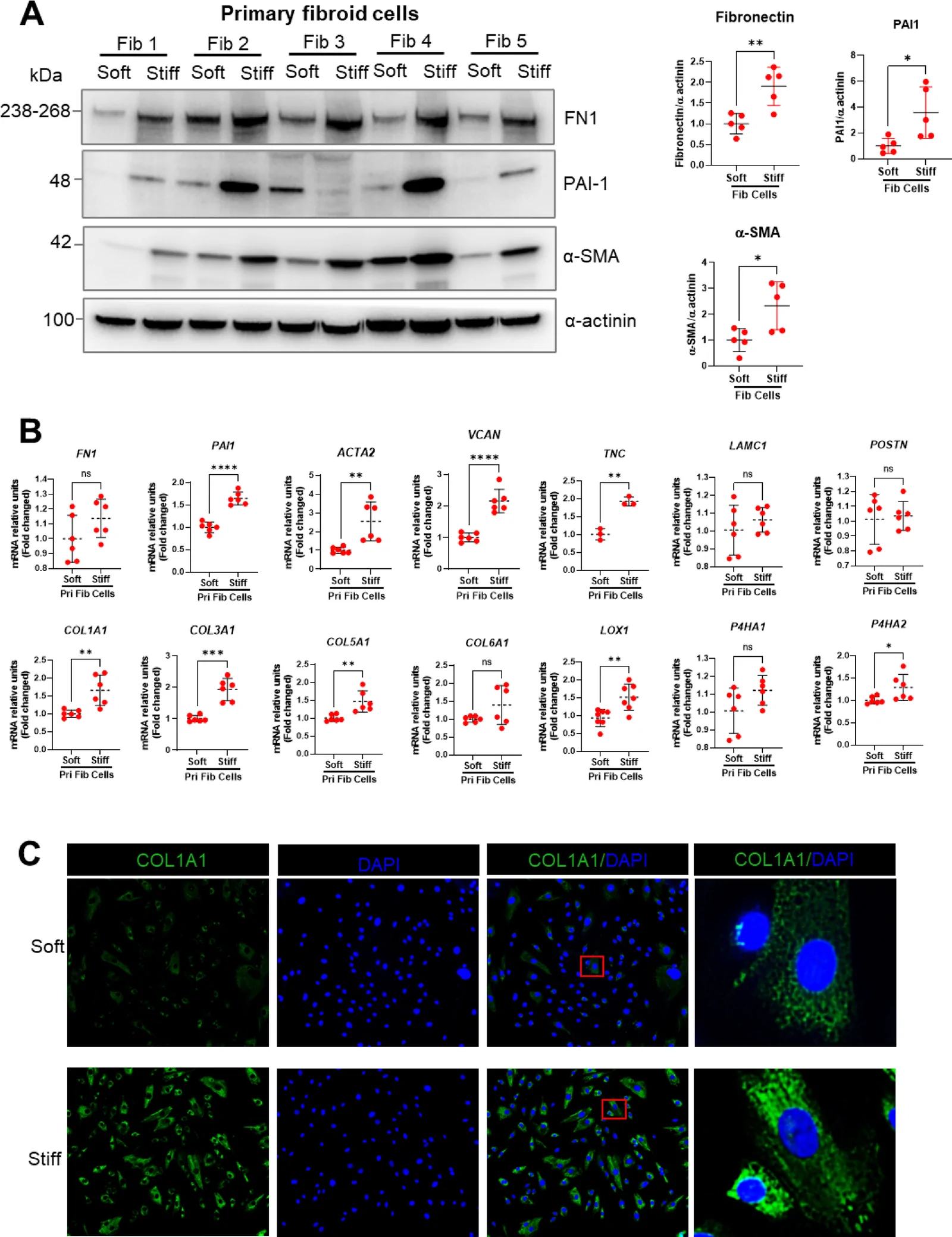
Substrate stiffness altered expression of fibrosis-associated proteins in cultured uterine fibroid cells. (**A**) Western blot images and quantification of fibronectin (FN1), PAI1, and α-SMA in primary fibroid cells cultured on a soft (930 kPa) and a stiff substrate (3 GPa) for 48 h (soft = 5, stiff = 5 from independent cultures of 5 fibroids isolated from 5 different patients) presented in scatter plots. Full immunoblots related to Fig. 2 are shown in Supplementary File 2, Fig. S2. Membranes were cut into several pieces (based on the molecular weight of proteins of interest) prior to hybridization with primary antibodies (FN1, PAI1, and α-SMA, and α-actinin) during western blot. MW (in kDa) are indicated. The α-actinin was used as a loading control to normalize the protein expression data. (**B**) Scatter plots showing the mRNA levels of fibrosis-associated proteins in primary fibroid cells cultured on a soft and a stiff substrate for 24 h (soft = 6–7, stiff = 6–7 replicates from independent cultures of two patients). *ARF1* was used as a housekeeping gene to normalize the gene expression data. (**C**) Representative images of immunofluorescence staining (COL1A1) from four biological replicates in primary fibroid cells cultured on soft and stiff substrates for 48 h. Shapiro–Wilk test was used to assess whether data were normally distributed. *p < 0.05; **p < 0.01; ***p < 0.001; ****p < 0.0001. Unpaired t test (**A**, **B**).

Uterine fibroids are characterized by excessive deposition of extracellular matrix (ECM) components, including collagens, fibronectin, and proteoglycans, which contribute to their dense, mechanically stiff microenvironment^7,36,37^. To investigate whether stiffness directly promotes the fibrotic cell phenotype, we quantified protein and mRNA levels of important fibrosis-associated markers in primary fibroid cells cultured on soft (930 kPa) or stiff (~ 3 GPa) substrates. Western analysis showed that stiff culture conditions significantly increased fibronectin (1.9-fold), plasminogen activator inhibitor-1 (PAI-1; 3.6-fold), and α-smooth muscle actin (α-SMA; 2.3-fold) protein levels compared to soft substrates (Fig. 2A). While fibronectin protein was elevated, *FN1* mRNA expression was unchanged (Fig. 2B). In contrast, mRNA levels of PAI-1 (*SERPINE1*; 1.7-fold) and α-SMA (*ACTA2*; 2.6-fold) mirrored the protein expression patterns (Fig. 2B). We next examined additional fibrosis-associated genes. Laminin C1 (*LAMC1*) and periostin (*POSTN*) levels were unchanged, whereas versican (*VCAN*) and tenascin C (*TNC*) mRNA were significantly upregulated (2.2-fold and 1.9-fold, respectively; Fig. 2B). Collagen subtypes *COL1A1* (1.7-fold), *COL3A1* (1.9-fold), and *COL5A1* (1.5-fold) were also elevated, while *COL6A1* remained unchanged (Fig. 2B). Immunofluorescence showed increased COL1A1 protein in fibroid cells cultured on stiff substrates (Fig. 2C). Next, we measured regulators of collagen maturation and cross-linking. *LOX1* (lectin-like oxidized low-density lipoprotein receptor 1), an enzyme critical for collagen accumulation^38^, was upregulated 1.5-fold, and *P4HA2* (prolyl 4-hydroxylase subunit alpha 2), involved in collagen biosynthesis^39^, was increased 1.3-fold, whereas *P4HA1* expression was unchanged (Fig. 2B). Together, these results demonstrate that substrate stiffness enhances the expression of fibrosis-associated factors in fibroid cells, supporting its role as a regulator of fibrotic remodeling in the fibroid microenvironment.

### Effect of substrate stiffness on mechanotransduction pathways in cultured uterine fibroid cells

**Fig. 3:**
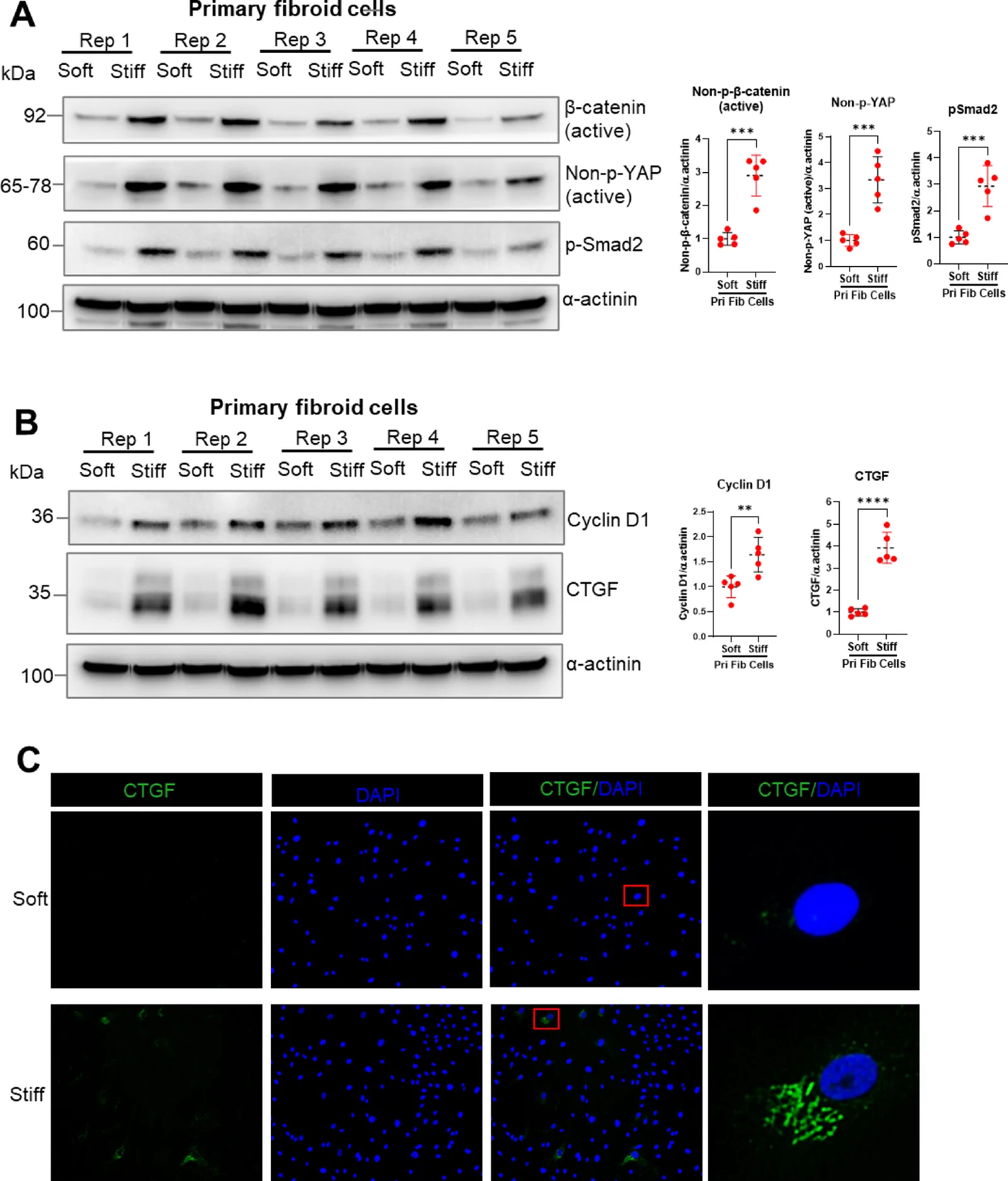
Increased substrate stiffness was associated with activation of pathways favoring growth in cultured uterine fibroid cells. (**A**) Western blot images (left) and quantification (right) of β-catenin (active), non-p-YAP (active), and phospho-Smad2 in primary fibroid cells cultured on a soft (930 kPa) and a stiff substrate (3 GPa) for 48 h (soft = 5, stiff = 5 from independent cultures) presented in scatter plots. (**B**) Western blot images (left) and quantification (right) of cyclin D1 and CTGF in primary fibroid cells cultured on a soft (930 kPa) and a stiff substrate (3 GPa) for 48 h (soft = 5, stiff = 5 from independent cultures) presented in scatter plots. Full immunoblots related to Fig. 3 are shown in Supplementary File 2, Figs. S3. Membranes were cut into several pieces (based on the molecular weight of proteins of interest) prior to hybridization with primary antibodies (β-catenin (active), non-p-YAP (active), and phospho-Smad2, cyclin D1, CTGF, and α-actinin) during western blot. MW (in kDa) are indicated. The α-actinin was used as a loading control to normalize the protein expression data. (**C**) Representative images of immunofluorescence staining (CTGF) from four biological replicates in primary fibroid cells cultured on soft and stiff substrates. Shapiro–Wilk test was used to assess whether data were normally distributed. *p < 0.05; **p < 0.01; ***p < 0.001; ****p < 0.0001. Unpaired t test (**A**, **B**).

Since substrate stiffness enhanced the expression of senescence- and fibrosis-associated markers in fibroid cells (Figs. 1 and 2), we next investigated the underlying mechanotransduction pathways. Primary fibroid cells were cultured on soft or stiff substrates, and levels of active β-catenin^40^, active (non-phosphorylated) YAP^6,28^, and phosphorylated-Smad2^41,42^, were quantified using western blot. Fibroid cells cultured on stiff substrates exhibited increased levels of active β-catenin (2.9-fold), active YAP (3.3-fold), and phosphorylated-Smad2 (2.9-fold) (Fig. 3A), indicating the activation of these pathways. To confirm downstream activation, we measured cyclin D1, a β-catenin/YAP target^43^, which was elevated 1.6-fold, and connective tissue growth factor (CTGF), a direct YAP and TGF-β/Smad target implicated in fibrosis^44,45^, which increased 3.9-fold (Fig. 3B). Immunofluorescence analysis further confirmed increased CTGF expression in fibroid cells cultured on stiff substrates (Fig. 3C). These findings indicate that mechanical stiffness activates β-catenin, YAP, and Smad2 signaling pathways, which may collectively drive the senescence–fibrotic phenotype in uterine fibroid cells.

### Dasatinib reduced senescence and fibrotic phenotypes in cultured uterine fibroid cells

**Fig. 4:**
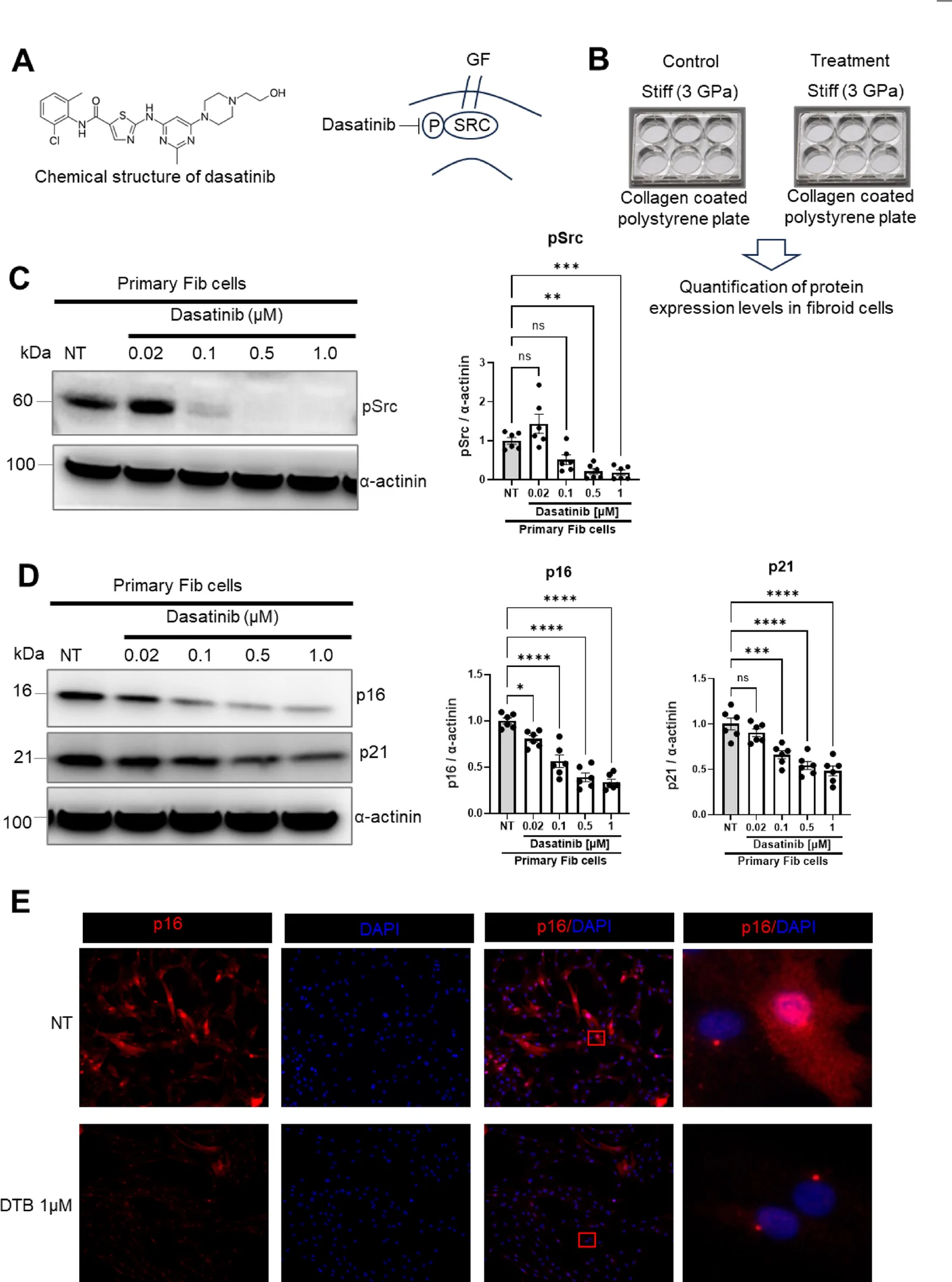
Dasatinib reduced expression of senescence markers in cultured uterine fibroid cells. (**A**) Chemical structure and diagram of inhibitory action of dasatinib against Src. (**B**) Schematic diagram of experimental design for quantification of protein expression levels from cultured fibroid cells cultured on a stiff substrate (3 GPa) and treated with dasatinib or control (DMSO) for 48 h. (**C**) Western blot images (left panel) and quantification (right panel) of phospho-Src in primary fibroid cells cultured on a stiff substrate and treated with dasatinib at 0.02, 0.1, 0.5 and 1 µM or control (DMSO) for 48 h (control = 6, dasatinib = 6 replicates from independent cultures). (**D**) Western blot images (left) and quantification (right) of p16 and p21 in primary fibroid cells cultured on a stiff substrate and treated with dasatinib at 0.02, 0.1, 0.5 and 1 µM or control (DMSO) for 48 h (control = 6, dasatinib = 6 replicates from independent cultures). Full immunoblots related to Fig. 4 are shown in Supplementary File 2, Fig. S4. Membranes were cut into several pieces (based on the molecular weight of proteins of interest) prior to hybridization with primary antibodies (phospho-Src, p16, p21, and α-actinin) during western blot. MW (in kDa) are indicated. The α-actinin was used as a loading control to normalize the protein expression data. (**E**) Representative images of immunofluorescence staining (p16) from four biological replicates in primary fibroid cells treated with dasatinib at 1 µM or control (DMSO) for 48 h. Shapiro–Wilk test was used to assess whether data were normally distributed. *p < 0.05; **p < 0.01; ***p < 0.001; ****p < 0.0001. One-way ANOVA (**C**, **D**).

These findings (Figs. 1, 2 and 3) led us to evaluate dasatinib, a senolytic compound approved for pediatric use in Philadelphia chromosome-positive chronic myeloid leukemia (CML)^46^, for its effects on fibroid cells exhibiting senescence and fibrotic phenotypes. Dasatinib is a potent Src inhibitor^47^ (Fig. 4A). Src, a non-receptor tyrosine kinase, regulates fibroblast-ECM interactions in some tissues^48^. To assess the specificity of dasatinib in this experiment, fibroid cells were treated with dasatinib at concentrations of 0.02, 0.1, 0.5, and 1 µM for 48 h, and phospho-Src levels were quantified (Fig. 4B). A significant reduction (78.9–81.7%) in phospho-Src levels was observed at dasatinib concentrations of 0.1, 0.5, and 1 µM (Fig. 4C), confirming its specificity. Next, we assessed the effects of dasatinib on senescence markers. Western blot analysis showed a dose-dependent significant reduction in p16 (19.5–66.2%) and p21 (33.9–51.7%) protein levels in fibroid cells compared to untreated controls (Fig. 4D). Immunofluorescence confirmed reduced p16 and p21 expression in dasatinib-treated fibroid cells (Fig. 4E, Supplementary File 1, Fig. S3A), supporting dasatinib’s senolytic activity. Additionally, a dose-dependent decrease in cell viability was observed in fibroid cells treated with dasatinib (0.1–2 µM) for both 24 and 48 h, as assessed by MTS assay (Supplementary File 1, Fig. S3B). These findings suggest that dasatinib reduces senescence marker expression and impairs the survival of senescent fibroid cells, consistent with its senolytic properties.

**Fig. 5:**
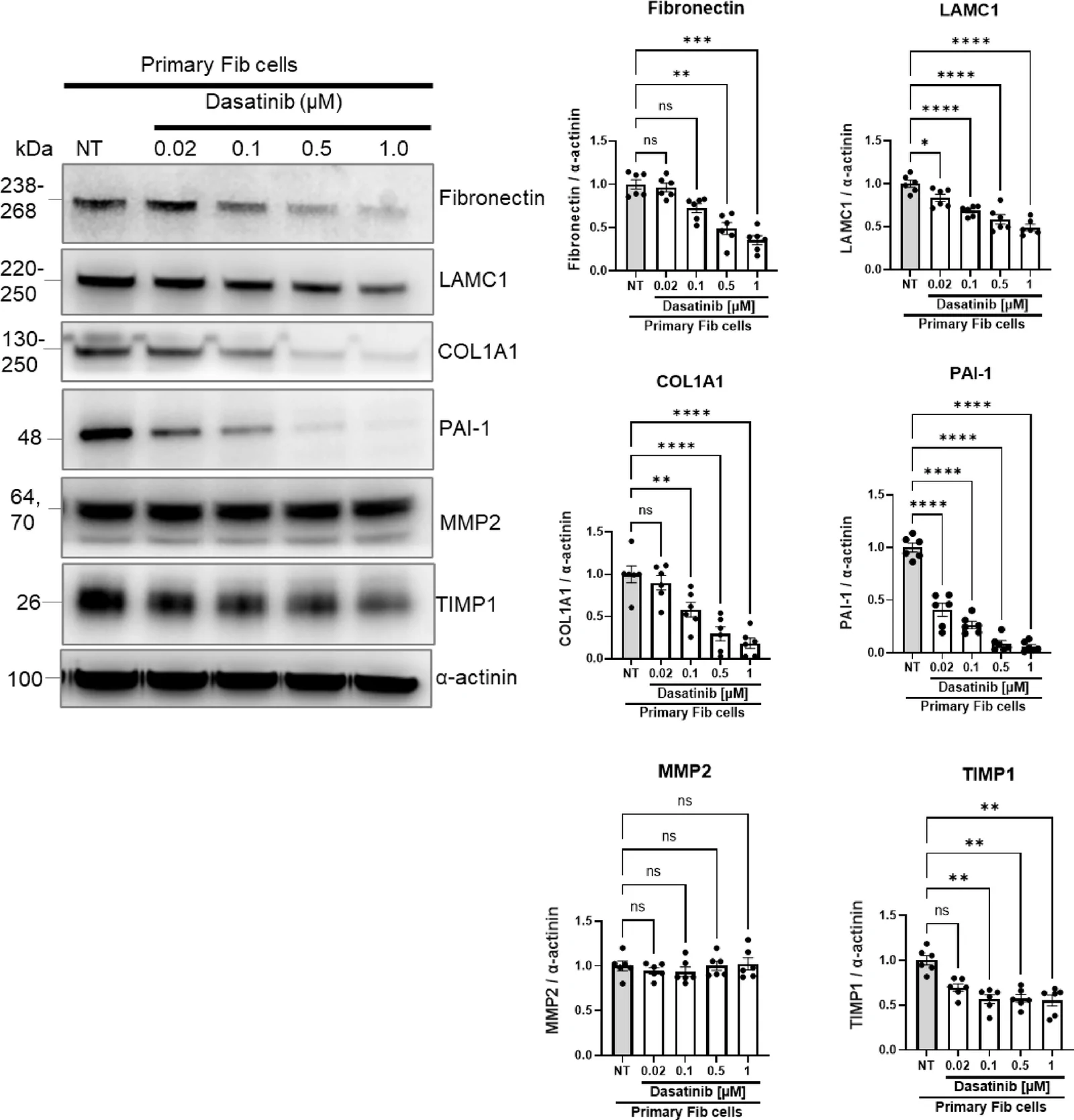
Dasatinib reduced expression of fibrosis-associated proteins in cultured uterine fibroid cells. Western blot images (left panel) and quantification (right) of fibronectin (FN1), LAMC1, COL1A1, PAI-1, MMP2, and TIMP1 in primary fibroid cells cultured a stiff substrate (3 GPa) and treated with dasatinib at 0.02, 0.1, 0.5 and 1 µM or control (DMSO) for 48 h (control = 6, dasatinib = 6 replicates from independent cultures). Full immunoblots related to Fig. 5 are shown in Supplementary File 2, Fig. S5. Membranes were cut into several pieces (based on the molecular weight of proteins of interest) prior to hybridization with primary antibodies (FN1, LAMC1, COL1A1, PAI-1, MMP2, and TIMP1, and α-actinin) during western blot. MW (in kDa) are indicated. The α-actinin was used as a loading control to normalize the protein expression data. Shapiro–Wilk test was used to assess whether data were normally distributed. *p < 0.05; **p < 0.01; ***p < 0.001; ****p < 0.0001. One-way ANOVA or Kruskal–Wallis test.

Since cellular senescence is closely linked to fibrotic conditions^49,50^, we tested the possibility that dasatinib would alter ECM remodeling in fibroid cells. Primary fibroid cell cultures were treated with dasatinib at concentrations of 0.02, 0.1, 0.5, and 1 µM for 48 h, and protein levels of fibrotic markers were quantified. Dasatinib treatment led to a dose-dependent significant reduction in ECM proteins, including fibronectin (51.0–64.5%), laminin C1 (16.4–50.8%), and collagen I (47.3–81.4%) (Fig. 5). Furthermore, dasatinib reduced PAI-1 protein levels (59.2–94.1%) in fibroid cells (Fig. 5). Although MMP2 levels remained unchanged, TIMP1 protein levels decreased (42.4–44.9%) following dasatinib treatment (Fig. 5). These data suggest that dasatinib effectively reduced fibrosis-associated protein expression in fibroid cells.

### Dasatinib reduced β-catenin signaling pathway in cultured uterine fibroid cells

**Fig. 6:**
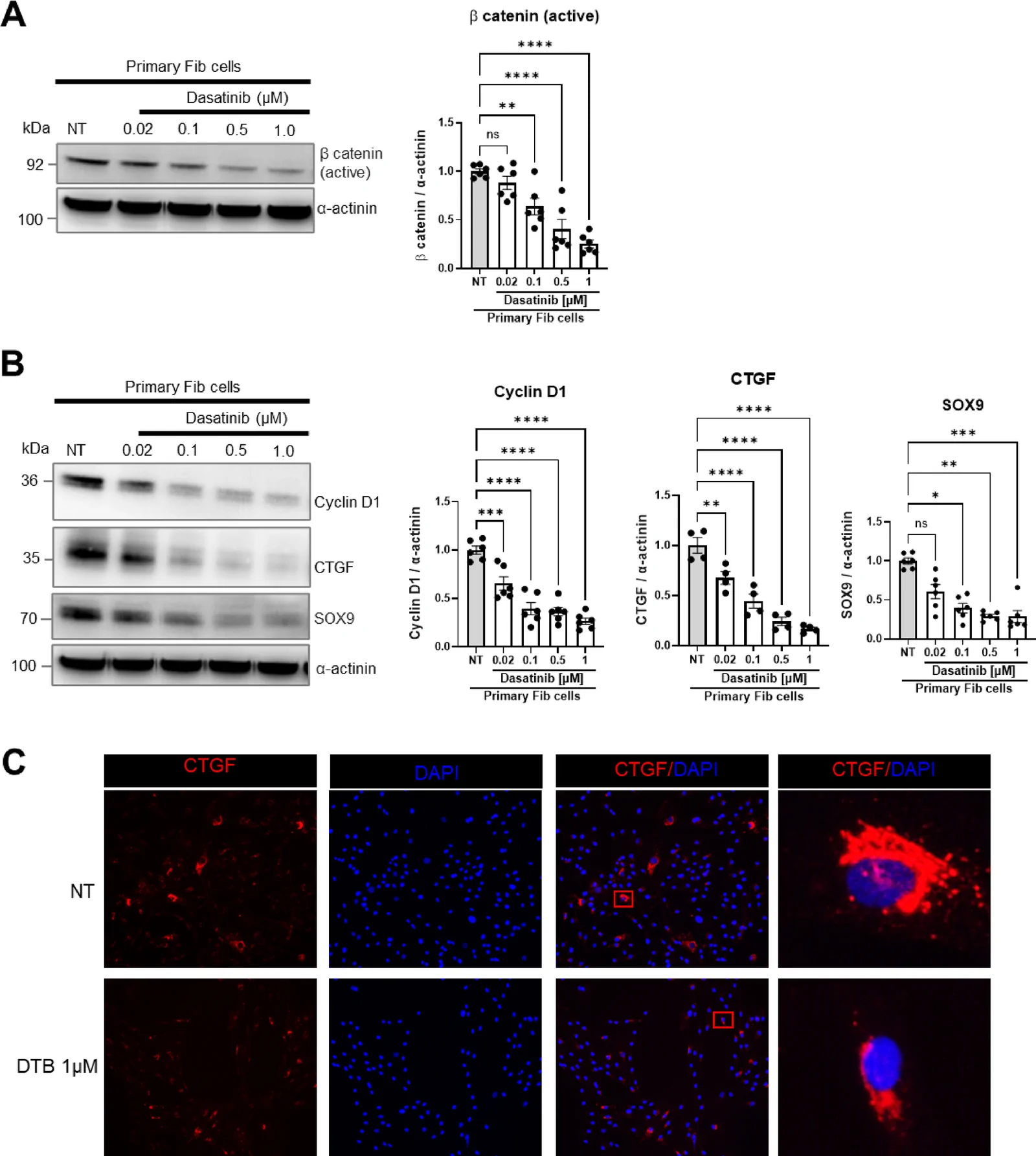
Dasatinib reduced the expression of β-catenin signaling pathway in cultured uterine fibroid cells. (**A**) Western blot images (left panel) and quantification (right) of β-catenin (active) in primary fibroid cells cultured a stiff substrate (3 GPa) and treated with dasatinib at 0.02, 0.1, 0.5 and 1 µM or control (DMSO) for 48 h (control = 6, dasatinib = 6 replicates from independent cultures). (**B**) Western blot images (left panel) and quantification (right) of β-catenin/YAP/Smad2-responsive genes cyclin D1, CTGF, and SOX9 in primary fibroid cells cultured a stiff substrate and treated with dasatinib at 0.02, 0.1, 0.5 and 1 µM or control (DMSO) for 48 h (control = 6, dasatinib = 6 replicates from independent cultures). Full immunoblots related to Fig. 6 are shown in Supplementary File 2, Fig. S6. Membranes were cut into several pieces (based on the molecular weight of proteins of interest) prior to hybridization with primary antibodies (β-catenin, non-p-YAP, and phospho-Smad2, cyclin D1, CTGF, SOX9, and α-actinin) during western blot. MW (in kDa) are indicated. The α-actinin was used as a loading control to normalize the protein expression data. (**C**) Representative images of immunofluorescence staining (CTGF) from four biological replicates in primary fibroid cells cultured a stiff substrate and treated with dasatinib at 1 µM or control (DMSO) for 48 h. Shapiro–Wilk test was used to assess whether data were normally distributed. *p < 0.05; **p < 0.01; ***p < 0.001; ****p < 0.0001. One-way ANOVA or Kruskal–Wallis test (**A**, **B**).

Next, building on the observation that WNT/β-catenin signaling can contribute to cellular senescence in a context-dependent manner^51,52^ and that active β-catenin is elevated in fibroid cells on stiff substrates (Fig. 3), we investigated the impact of dasatinib on β-catenin signaling. Fibroid cells were treated with dasatinib at concentrations of 0.02, 0.1, 0.5, and 1 µM for 48 h, and levels of active β-catenin were measured using western blot. Dasatinib treatment resulted in a dose-dependent reduction in active β-catenin (36.2–74.6%) (Fig. 6A). Additionally, dasatinib decreased the expression of β-catenin-responsive genes. Cyclin D1 protein levels were reduced by 34.5–73.4%, and CTGF protein levels decreased by 32.5–83.5% following dasatinib treatment (Fig. 6B). Immunofluorescence staining confirmed a reduction in CTGF protein expression in dasatinib-treated fibroid cells (Fig. 6C). We also examined SOX9, a transcription factor that interacts with TCF and modulates WNT/β-catenin signaling in a context-dependent manner^53^, which was elevated in fibroid relative to myometrial cells (Supplementary File 1, Fig. S4). SOX9 protein levels were significantly reduced (60.4–71.3%) in dasatinib-treated fibroid cells (Fig. 6B). These findings demonstrate that dasatinib suppressed β-catenin signaling, which may help explain its effects on reducing senescence and fibrotic phenotypes in fibroid cells. Since dasatinib targets Src, the results also suggest that Src may contribute to β-catenin activation and the development of the senescence-fibrotic phenotype in uterine fibroid cells.

### Inhibition of β-catenin by ICG-001 is associated with reduced senescence-fibrotic phenotype in cultured uterine fibroid cells

**Fig. 7:**
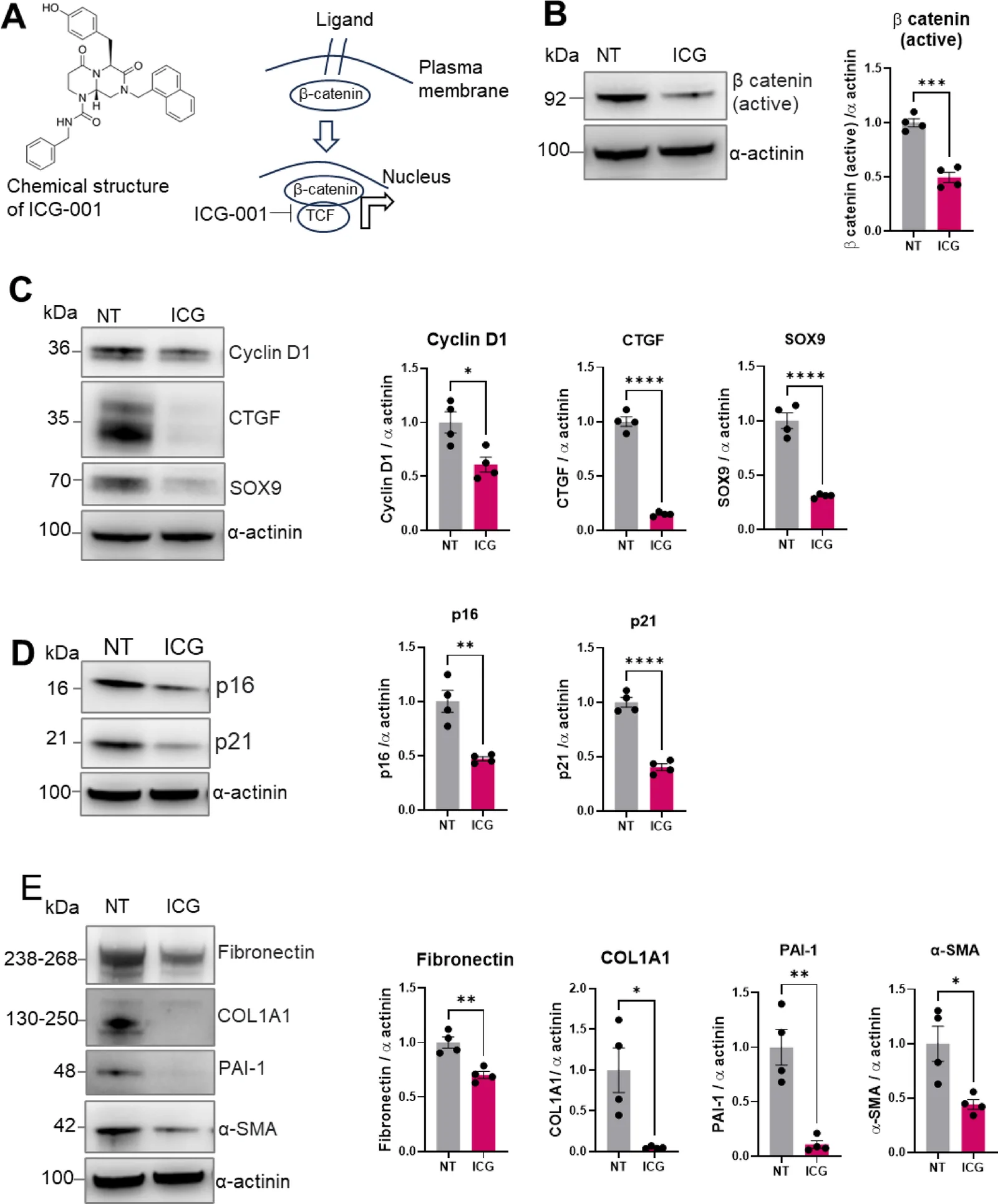
Inhibition of β-catenin by ICG-001 is associated with reduced senescence-fibrotic phenotype in cultured uterine fibroid cells. (**A**) Chemical structure and diagram of inhibitory action of ICG-001 against β-catenin. (**B**) Western blot images (left) and quantification (right) of β-catenin (active) in primary fibroid cells cultured on a stiff substrate (3 GPa) and treated with ICG-001 (2.5 µM) or control (DMSO) for 48 h (control = 4, ICG-001 = 4 replicates from independent cultures). (**C**) Western blot images (left panel) and quantification (right panel) of β-catenin-responsive genes cyclin D1, CTGF, and SOX9 in primary fibroid cells cultured a stiff substrate and treated with ICG-001 (2.5 µM) or control (DMSO) for 48 h (control = 4, ICG-001 = 4 replicates from independent cultures). (**D**) Western blot images (left panel) and quantification (right panel) of senescence markers (p16 and p21) in primary fibroid cells cultured a stiff substrate and treated with ICG-001 (2.5 µM) or control (DMSO) for 48 h (control = 4, ICG-001 = 4 replicates from independent cultures). (**E**) Western blot images (left panel) and quantification (right panel) of fibrosis-associated proteins (FN1, COL1A1, α-SMA, and PAI-1) in primary fibroid cells cultured a stiff substrate and treated with ICG-001 (2.5 µM) or control (DMSO) for 48 h (control = 4, ICG-001 = 4 replicates from independent cultures). Full immunoblots related to Fig. 7 are shown in Supplementary File 2, Fig. S7. Membranes were cut into several pieces (based on the molecular weight of proteins of interest) prior to hybridization with primary antibodies (β-catenin, cyclin D1, CTGF, SOX9, p16, p21, FN1, COL1A1, α-SMA, and PAI-1, and α-actinin) during western blot. MW (in kDa) are indicated. The α-actinin was used as a loading control to normalize the protein expression data. Shapiro–Wilk test was used to assess whether data were normally distributed. *p < 0.05; **p < 0.01; ***p < 0.001; ****p < 0.0001. Unpaired t test (**B**–**E**).

The contribution of β-catenin to fibroid pathogenesis remains is not fully understood^40^, particularly regarding the role of β-catenin in maintaining the senescence-fibrotic phenotype. To investigate this, we treated cells with ICG-001, a selective small-molecule inhibitor that disrupts the interaction between β-catenin and the co-activator CBP (CREB-binding protein), thereby blocking WNT/β-catenin–mediated transcription^54^ (Fig. 7A). Cell viability was assessed following 24 and 48-h ICG-001 treatment (0.5–20 µM) using the MTS assay. A significant reduction in fibroid cell viability was observed at concentrations of 1 µM and higher, compared to the untreated control (Supplementary File 1, Fig. S5). Western blot analysis confirmed effective pathway inhibition with 2.5 µM ICG-001, showing a 50.4% reduction in transcriptionally active β-catenin (Fig. 7B). Consistent with this, β-catenin–responsive targets cyclin D1 and CTGF were decreased (Fig. 7C). Furthermore, ICG-001 treatment for 48 h significantly reduced SOX9 protein levels (Fig. 7C), suggesting regulatory crosstalk between β-catenin and SOX9 in fibroid cells.

Importantly, ICG-001 treatment also decreased the expression of senescence markers p16 and p21 (Fig. 7D), implicating β-catenin in the regulation of senescence of fibroid cells. In the context of fibrosis, ICG-001 significantly decreased protein levels of ECM proteins, including fibronectin and COL1A1, as well as fibrotic marker PAI-1 (Fig. 7E). Additionally, α-SMA protein levels were significantly reduced (Fig. 7E), highlighting a potential role of β-catenin in the fibrotic phenotype of fibroid cells. These data are consistent with the conclusion that β-catenin signaling plays a role in maintaining the senescence-fibrotic phenotype of uterine fibroid cells.

### Inhibition of β-catenin by PRI-724 treatment reduced the senescence-fibrotic phenotype in cultured uterine fibroid cells

**Fig. 8:**
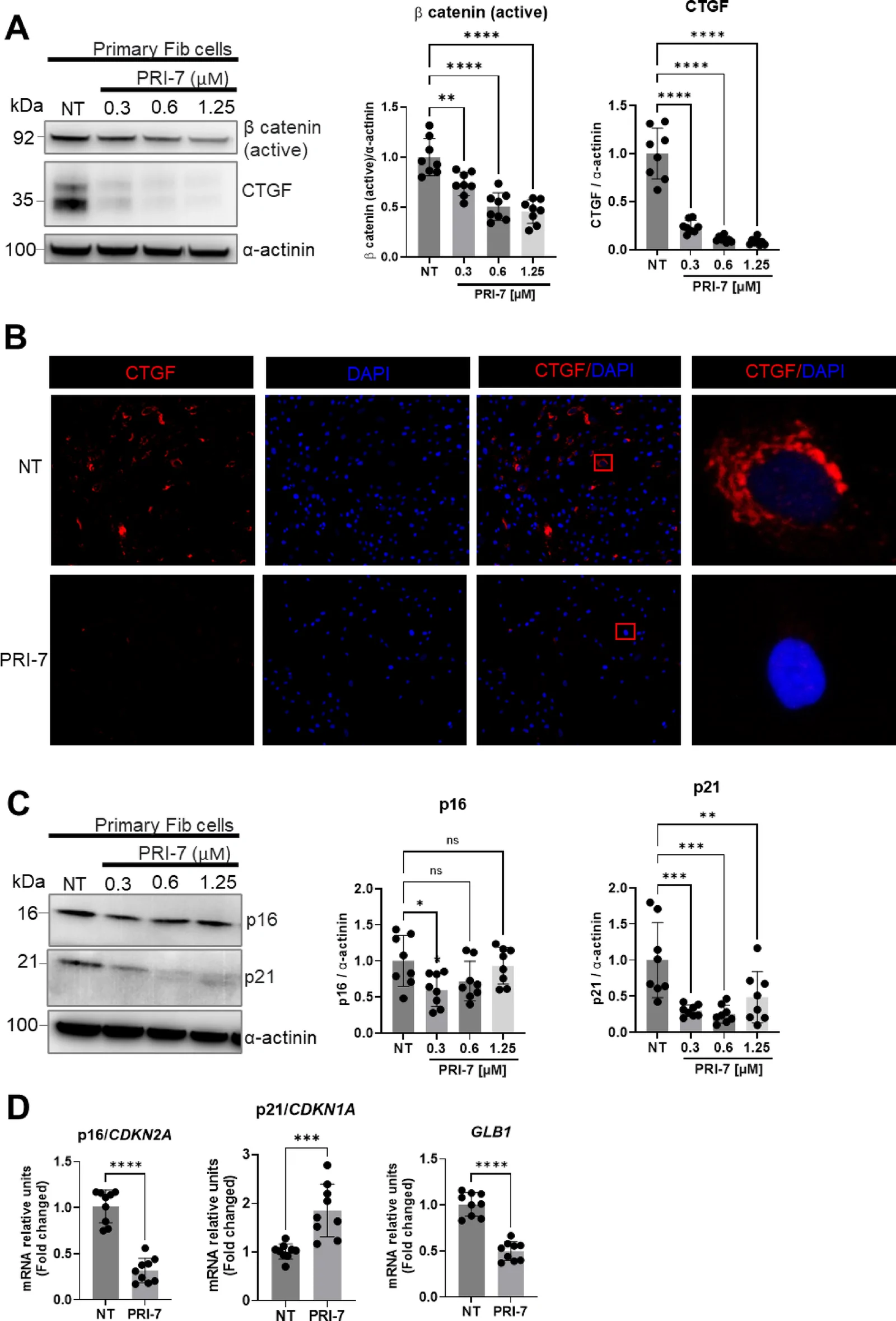
PRI-724-mediated inhibition of β-catenin reduces senescence in cultured uterine fibroid cells. (**A**) Western blot images (left) and quantification (right) of β-catenin (active) and CTGF in primary fibroid cells cultured on a stiff substrate (3 GPa) and treated with PRI-724 at 0.3, 0.6, and 1.25 µM or control (DMSO) for 48 h (control = 8, PRI-7 = 8 replicates from independent cultures). (**B**) Representative images of immunofluorescence staining (CTGF) from four biological replicates in primary fibroid cells cultured on a stiff substrate and treated with PRI-7 at 1.25 µM or control (DMSO) for 48 h. (**C**) Western blot images (left panel) and quantification (right panel) of p16 and p21 in primary fibroid cells cultured on a stiff substrate and treated with PRI-724 at 0.3, 0.6, and 1.25 µM or control (DMSO) for 48 h (control = 8, PRI-7 = 8 replicates from independent cultures). Full immunoblots related to Fig. 8 are shown in Supplementary File 2, Fig. S8. Membranes were cut into several pieces (based on the molecular weight of proteins of interest) prior to hybridization with primary antibodies (β-catenin, CTGF, p16, p21, and α-actinin) during western blot. MW (in kDa) are indicated. The α-actinin was used as a loading control to normalize the protein expression data. (**D**) Scatter plots showing the mRNA levels of senescence-associated proteins in primary fibroid cells cultured on a stiff substrate and treated with PRI-724 at 1.25 µM or control (DMSO) for 48 h (NT = 9, PRI-7 = 9 replicates from independent cultures). *ARF1* was used as a housekeeping gene to normalize the gene expression data. Shapiro–Wilk test was used to assess whether data were normally distributed. *p < 0.05; **p < 0.01; ***p < 0.001; ****p < 0.0001. One-way ANOVA (**A**, **C**), Unpaired t test (**D**).

To further evaluate the role of β-catenin in maintaining the senescence-fibrotic phenotype in uterine fibroid cells, we tested the effects of foscenvivint (PRI-724), a small-molecule inhibitor currently in clinical trials that selectively disrupts the interaction between β-catenin and its coactivator CBP, thereby blocking Wnt/β-catenin transcriptional activity^55^ (Supplementary File 1, Fig. S6A). Fibroid cell viability was assessed by MTS assay following treatment with increasing concentrations of PRI-724 (0.156–2.5 µM) for 24 and 48 h. A dose-dependent decrease in cell viability was observed, with significant reductions at concentrations of 0.625 μM and higher at both time points compared to non-treated controls (Supplementary File 1, Fig. S6B). To examine the molecular effects of β-catenin inhibition, primary fibroid cells were treated with 0.3–1.25 µM PRI-724 for 48 h, and expression of important markers of senescence-fibrotic phenotype was analyzed by western blot, immunofluorescence, and qPCR. Western blot analysis showed a dose-dependent reduction in β-catenin protein levels (26.9–54.6%) following PRI-724 treatment (Fig. 8A), confirming pathway inhibition. This was accompanied by a pronounced decrease in the downstream target CTGF, with protein levels reduced by 76.1–91.8% (Fig. 8A). Immunofluorescence analysis further supported the suppression of CTGF protein expression after 1.25 µM treatment (Fig. 8B), signifying a marked suppression of Wnt/β-catenin signaling.

Senescence markers were also affected by treatment with PRI-724, with p16 protein levels decreasing by 40.4% (at 0.3 µM) and p21 protein levels by 51.5–70% (0.3 to 1.25 µM) (Fig. 8C). At the mRNA level, p16 (*CDKN2A*) expression was reduced by 68%, while p21 (*CDKN1A*) showed a 1.9-fold increase (Fig. 8D). Immunofluorescence analysis showed reduced nuclear p21 expression in uterine fibroid cells following treatment, as indicated by reduced green fluorescence intensity compared to the control (Supplementary File 1, Fig. S6C). In addition, β-galactosidase (*GLB1*) mRNA levels were reduced by 50% at 1.25 µM (Fig. 8D). These findings suggest that PRI-724 modulates senescence phenotype in fibroid cells.

**Fig. 9:**
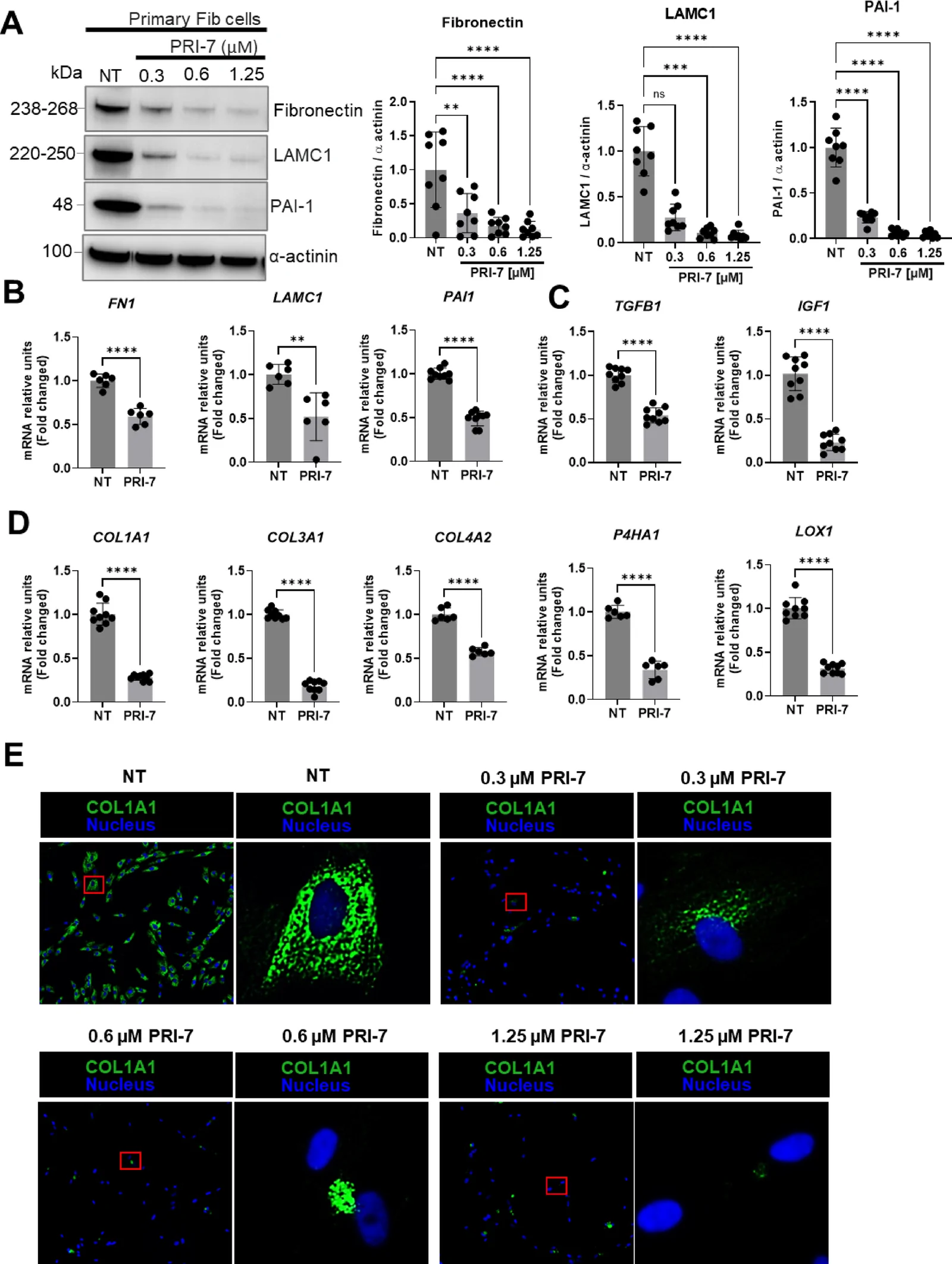
PRI-724 treatment reduces fibrotic phenotype in cultured uterine fibroid cells. (**A**) Western blot images (left) and quantification (right) of fibrosis-associated proteins in primary fibroid cells cultured on a stiff substrate (3 GPa) and treated with PRI-724 at 0.3, 0.6, and 1.25 µM or control (DMSO) for 48 h (control = 8, PRI-7 = 8 replicates from independent cultures). Full immunoblots related to Fig. 9 are shown in Supplementary File 2, Fig. S9. Membranes were cut into several pieces (based on the molecular weight of proteins of interest) prior to hybridization with primary antibodies (fibronectin, LAMC1, PAI-1, and α-actinin) during western blot. MW (in kDa) are indicated. The α-actinin was used as a loading control to normalize the protein expression data. (**B**–**D**) Scatter plots showing the mRNA levels of fibrosis-associated genes in primary fibroid cells cultured on a stiff substrate and treated with PRI-724 at 1.25 µM or control (DMSO) for 48 h (NT = 6–9, PRI-7 = 6–9 replicates from independent cultures). *ARF1* was used as a housekeeping gene to normalize the gene expression data. (**E**) Representative immunofluorescence images of COL1A1 in primary fibroid cells cultured on a stiff substrate for 48 h following treatment with PRI-7 at 0.3, 0.6, and 1.25 µM or control (DMSO) for 48 h. Shapiro–Wilk test was used to assess whether data were normally distributed. *p < 0.05; **p < 0.01; ***p < 0.001; ****p < 0.0001; One-way ANOVA or Kruskal–Wallis test (**A**); Unpaired t test (**B**–**D**).

To further assess the fibrotic phenotype, we quantified ECM-related genes and proteins in cultures treated with PRI-724. FN1 mRNA and protein levels were reduced by 41% and 63.7–87.6%, respectively, and LAMC1 expression decreased by 48% (mRNA) and 89.6–91.8% (protein) (Fig. 9A,B). FN1 suppression was confirmed by immunofluorescence (Supplementary File 1, Fig. S7). Similarly, PAI-1 protein and mRNA levels were significantly reduced by 76.7–95.6% and 51%, respectively (Fig. 9A,B). PRI-724 also was associated with reduced expression of fibrotic growth factors *TGFB1* (46%) and *IGF1* (77%) (Fig. 9C). Furthermore, collagen-related genes showed marked reduction. *COL1A1*, *COL3A1*, *COL4A2*, *P4HA1* (collagen synthesis enzyme), and *LOX1* (collagen cross-linking enzyme) were all significantly downregulated, with *LOX1* and *P4HA1* reduced by 68% and 66%, respectively (Fig. 9D). Immunofluorescence staining supported reduced COL1A1 protein levels following PRI-724 treatment (Fig. 9E). Overall, these findings demonstrate that pharmacological inhibition of the β-catenin/CBP complex by PRI-724 reduced both senescence- and fibrosis-associated markers in uterine fibroid cells. These results suggest that β-catenin signaling plays a key role in the induction or maintenance of the senescence-fibrotic phenotype in fibroid cells.

## Discussion

**Fig. 10:**
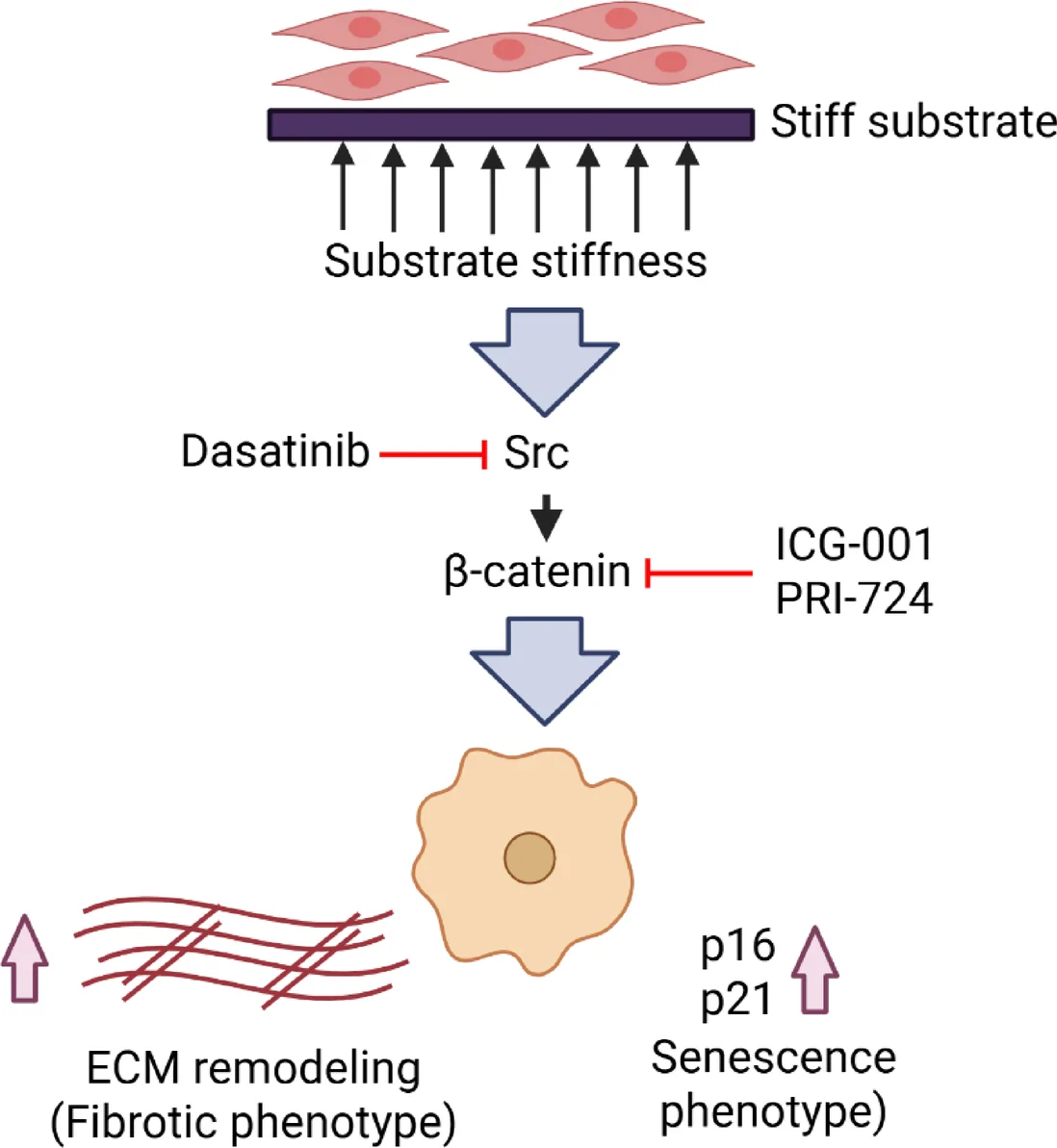
Substrate stiffness promotes a senescence-fibrotic phenotype in uterine fibroid cells via Src/β-catenin signaling. This schematic summarizes the proposed mechanism by which mechanical stiffness contributes to fibroid pathogenesis. Stiff substrates, mimicking the fibrotic microenvironment of uterine fibroids, activate Src and downstream β-catenin signaling. This activation may lead to increased expression of senescence markers (p16 and p21) and extracellular matrix (ECM) deposition, contributing to the senescence-fibrotic phenotype. Pharmacological inhibition of Src with dasatinib or disruption of β-catenin/CBP interaction using ICG-001 or PRI-724 alters the senescence-fibrotic phenotype in fibroid cells. Figure created in Biorender.com.

These results are consistent with a functional role for mechanical stiffness and senescent cells in the pathogenesis of uterine fibroids. Further, we identified Src and β-catenin signaling as regulators of the senescence and fibrotic phenotypes (Fig. 10). We demonstrated that senescent cells were elevated in fibroids relative to myometrium, and that substrate stiffness enhanced the expression of senescence and fibrosis associated markers. Mechanistically, pharmacological inhibition of Src and β-catenin signaling reduced the expression of both senescence and fibrosis markers, suggesting that these pathways may contribute to induction or the maintenance of senescence and fibrosis in fibroid cells (Fig. 10).

Although fibroid tumors are composed of multiple cell types, including smooth muscle cells, fibroblast, and endothelial cells^56,57^, the observed upregulation of p16 in fibroid compared to myometrial cells, along with prior reports of increased p16 and p14^ARF^ expression in fibroid tissues^18,19^, suggests involvement of senescent cells in fibroid pathogenesis. While the presence of senescent cells in fibroid tumors is evident, the mechanisms driving their accumulation and their functional contributions to fibroid growth had not been rigorously examined. Our data suggest that mechanical stiffness within the fibroid microenvironment promotes cellular senescence. This is evidenced by increased expression of p16 and p21 in fibroid cells cultured on stiff substrates. This is consistent with our earlier findings in dermal fibroblasts where stiffness induced sustained p16 and p21 expression over time^31^. Together, these observations suggest the mechanoregulation of cellular senescence in fibroid cells. Furthermore, this observation highlights the importance of additional studies to further characterize specific cell types that undergo senescence and to elucidate their roles in fibroid growth.

We also investigated the mechanoregulation of the fibrotic phenotype in fibroid cells. We found that fibroid cells cultured on stiff substrates exhibited increased expression of important ECM and fibrotic markers, including fibronectin, and PAI-1. Notably, the upregulation of collagen subtypes and *LOX1*, a mediator of collagen cross-linking, suggests the role of mechanical stiffness in promoting a fibrotic phenotype. This aligns with observations from other fibrotic and tumor contexts where increased tissue stiffness is associated with ECM deposition and remodeling^58^. Increased α-SMA expression further points to myofibroblast activation, a hallmark of fibrotic remodeling^59^.

At the signaling level, we observed that stiff substrate activates multiple mechanoresponsive pathways including β-catenin, YAP, and Smad along with their downstream targets, CTGF and cyclin D1. These pathways have been implicated in fibrotic and proliferative responses to mechanical cues. Our previous work established a link between ECM stiffness and YAP/Smad activation in fibroids^6^, and Ko et al. demonstrated that β-catenin signaling is similarly activated by stiff ECM in fibroid cells^11^. Together, these findings support a convergence of signaling pathways that coordinate cellular responses to mechanical stiffness in fibroid pathogenesis.

Dasatinib, a known senolytic compound^46^ and Src inhibitor^47^, demonstrated efficacy in reducing both senescence and fibrosis-associated markers in fibroid cells. The dose-dependent reduction in p16 and p21 protein levels confirmed the senolytic properties of dasatinib. Furthermore, the ability of dasatinib to downregulate fibrotic markers, including fibronectin, laminin C1, collagen I, and PAI-1, suggests its potential as an anti-fibrotic agent. Importantly, the inhibition of β-catenin signaling pathways by dasatinib, as evidenced by reduced levels of active β-catenin, and its downstream targets such as cyclin D1 and CTGF, provides a mechanistic basis for its effects on the senescence-fibrotic phenotype. Since dasatinib targets Src^47^, these findings suggest that Src functions as an upstream regulator of β-catenin activity. Previous studies have shown that activated Src promotes nuclear accumulation of β-catenin and enhances its transcriptional activity, leading to elevated expression of target genes such as cyclin D1^60^. Additionally, early activation of Src has been reported to influence cell fate by shifting the balance from apoptosis to senescence^61^. Together, these data support a model in which Src may facilitate β-catenin activation and the maintenance of a senescence-fibrotic phenotype in uterine fibroid cells.

The roles of signaling pathways such as YAP^6,28^ and Smad^41,42^ to promote fibrosis in fibroid cells have been described, yet the contribution of β-catenin to fibroid pathogenesis remains comparatively underexplored^40^. Specifically, the involvement of β-catenin in orchestrating the senescence-fibrotic phenotype in uterine fibroid cells has not been extensively investigated. Although WNT/β-catenin signaling is known to promote proliferation and inhibit senescence in epithelial cells and fibroblasts^51^, and is frequently associated with cancer progression^62^, emerging evidence suggests a more complex role. Li et al. reported that activation of WNT/β-catenin signaling increased markers of senescence, including SA-β Gal, p53, p16, and acetylated p53, suggesting a potential context dependent contribution to cellular senescence^51,52^. To study the involvement of β-catenin in the senescence-fibrotic phenotype in uterine fibroid cells, we used two pharmacological inhibitors of WNT/β-catenin signaling, ICG-001 and PRI-724^54,55^. Treatment with ICG-001 reduced the expression of senescence markers p16 and p21, as well as fibrosis-associated proteins, including fibronectin, COL1A1, and PAI-1, supporting a role for β-catenin in regulating the senescence-fibrotic axis. We also observed a decrease in SOX9 expression following β-catenin inhibition, suggesting additional regulatory complexity that warrants further investigation. Similarly, PRI-724 treatment significantly suppressed β-catenin protein levels and downstream targets such as CTGF, as well as senescence markers including p16, p21, and β-galactosidase. In addition, PRI-724 reduced the expression of important ECM components, fibrotic growth factors, and collagen-related enzymes. These complementary findings from ICG-001 and PRI-724 treatment illustrate the importance of β-catenin signaling as a regulator of senescence and fibrosis in uterine fibroids.

In conclusion, we observed a functional link between mechanical stiffness and the senescence and fibrotic phenotype in uterine fibroid cells. The identification of Src and β-catenin signaling as potential regulators of this phenotype provides a mechanistic framework for understanding fibroid pathogenesis. The ability of pharmacological inhibitors such as dasatinib, ICG 001, and PRI 724 to suppress senescence and fibrosis markers highlights these pathways as promising therapeutic targets for future investigation.

## Supplementary Information

Below is the link to the electronic supplementary material.

Supplementary Material 1

Supplementary Material 2

## Data Availability

All data associated with this study are present in the paper or the Supplementary Materials and are available from the corresponding authors on reasonable request.
