# Supplementary material for: Src and β-catenin control a senescence-fibrotic phenotype in uterine fibroid cells
Source: Sci Rep. 2026 Jun 26;16:29418. doi: 10.1038/s41598-026-57742-5 (PMC13594328; doi:10.1038/s41598-026-57742-5)
Supplement: Supplementary file 1 [file 41598_2026_57742_MOESM1_ESM.pdf]

Supplementary Info File 1

Fig. S1 PageRuler™ Plus Prestained Protein Ladder, 10 to 250 kDa. (A) An image sourced from the manufacturer, Thermo Fisher Scientific, showcasing the PageRuler™ Plus Prestained Protein Ladder. This ladder consists of nine prestained proteins (blue, orange, and green) ranging from 10 to 250 kDa, commonly used as molecular size standards in protein electrophoresis (SDS-PAGE) and western blotting (Thermo Fisher Scientific, cat no. 26619). **(B)** A representative image from our lab illustrating the use of the PageRuler™ Plus Prestained Protein Ladder. The ladder was run on a NuPage 4-12% Bis-Tris gel (Invitrogen by Thermo Fisher Scientific, cat no. NP0321BOX), and the proteins were transferred onto a nitrocellulose membrane filter paper with a 0.2 µM pore size (Invitrogen by Thermo Fisher Scientific, cat no. LC2000).

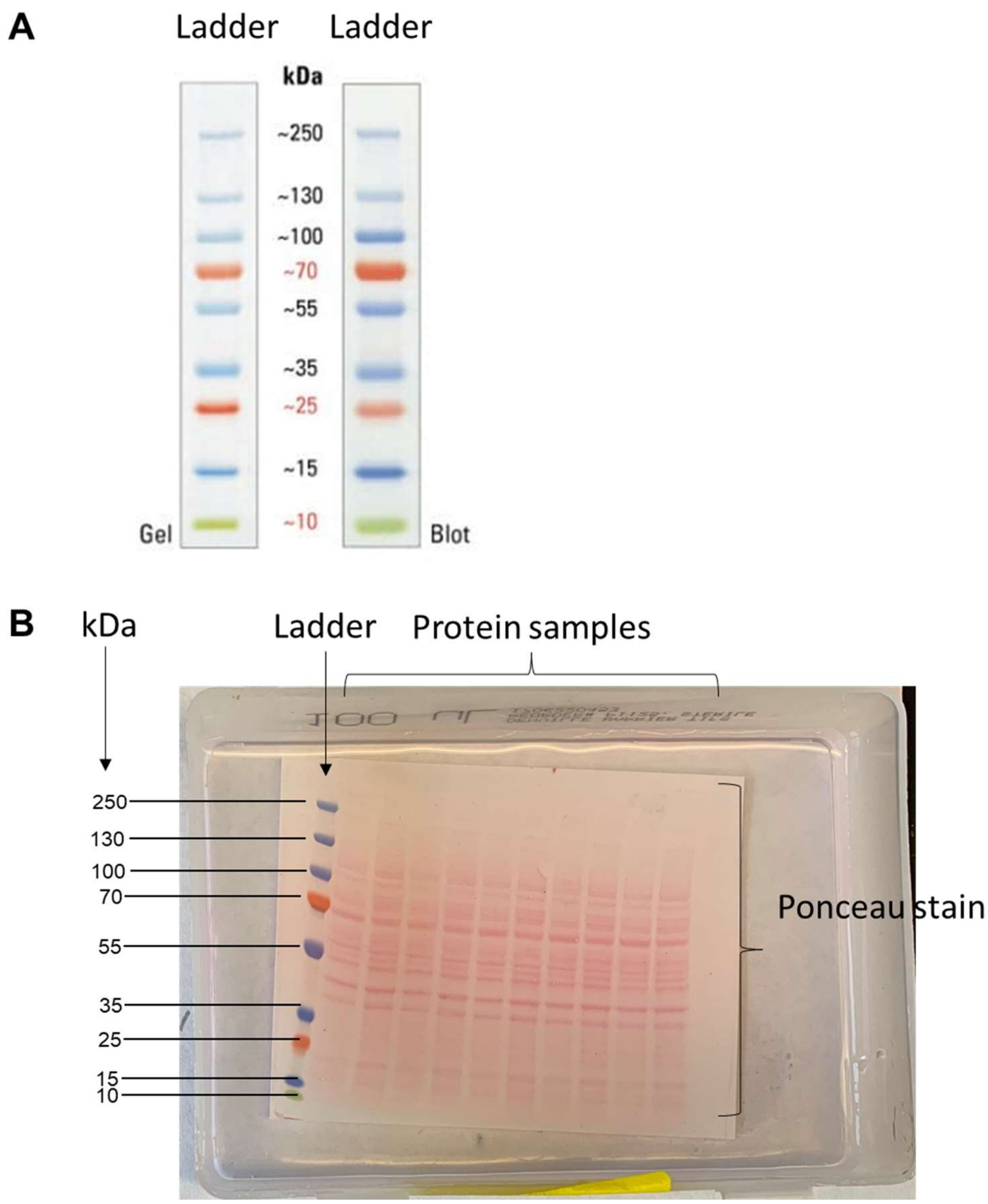

Fig. S2 The p21 expression in fibroid cells cultured on soft and stiff substrates. Representative 10X immunofluorescence images of p21 from four biological replicates of primary fibroid cells cultured on soft and stiff substrates for 5 days.

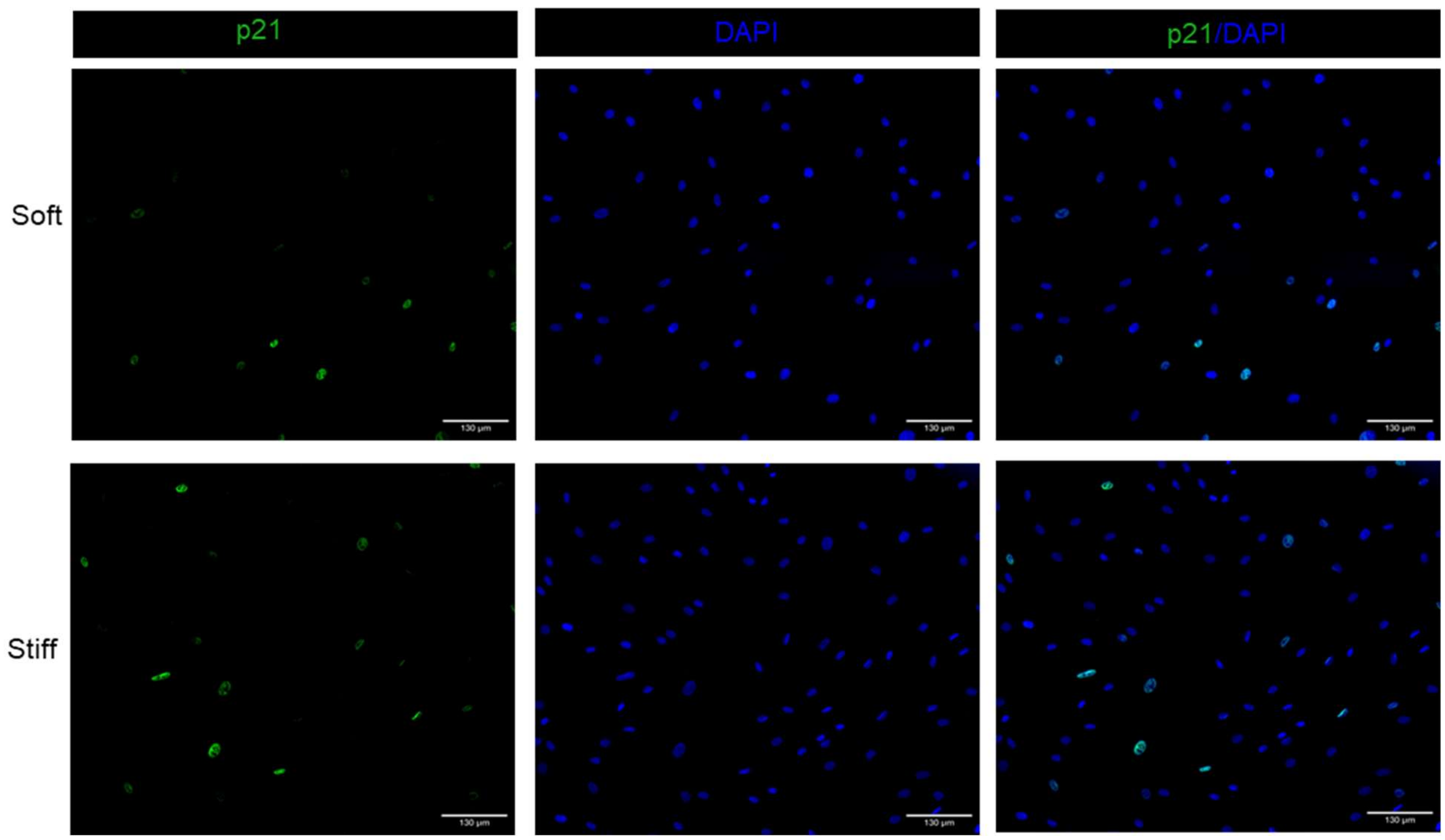

Fig. S3 Effect of dasatinib (DTB) on p21expression and cell viability in cultured uterine fibroid cells. A) Representative immunofluorescence images of p21 in fibroid cells from four biological replicates in fibroid cells treated with DTB at 1μM for 48 hours. **B**) Cell viability of fibroid cells was assessed by MTS assay following treatment with dasatinib at different concentrations for 24 and 48 hours (n=8 biological replicates). Shapiro-Wilk test was used to assess whether data are normally distributed or not. $^{*}p < 0.05$; $^{**}p < 0.01$; $^{***}p < 0.001$; $^{****}p < 0.0001$. One-way ANOVA (**B**).

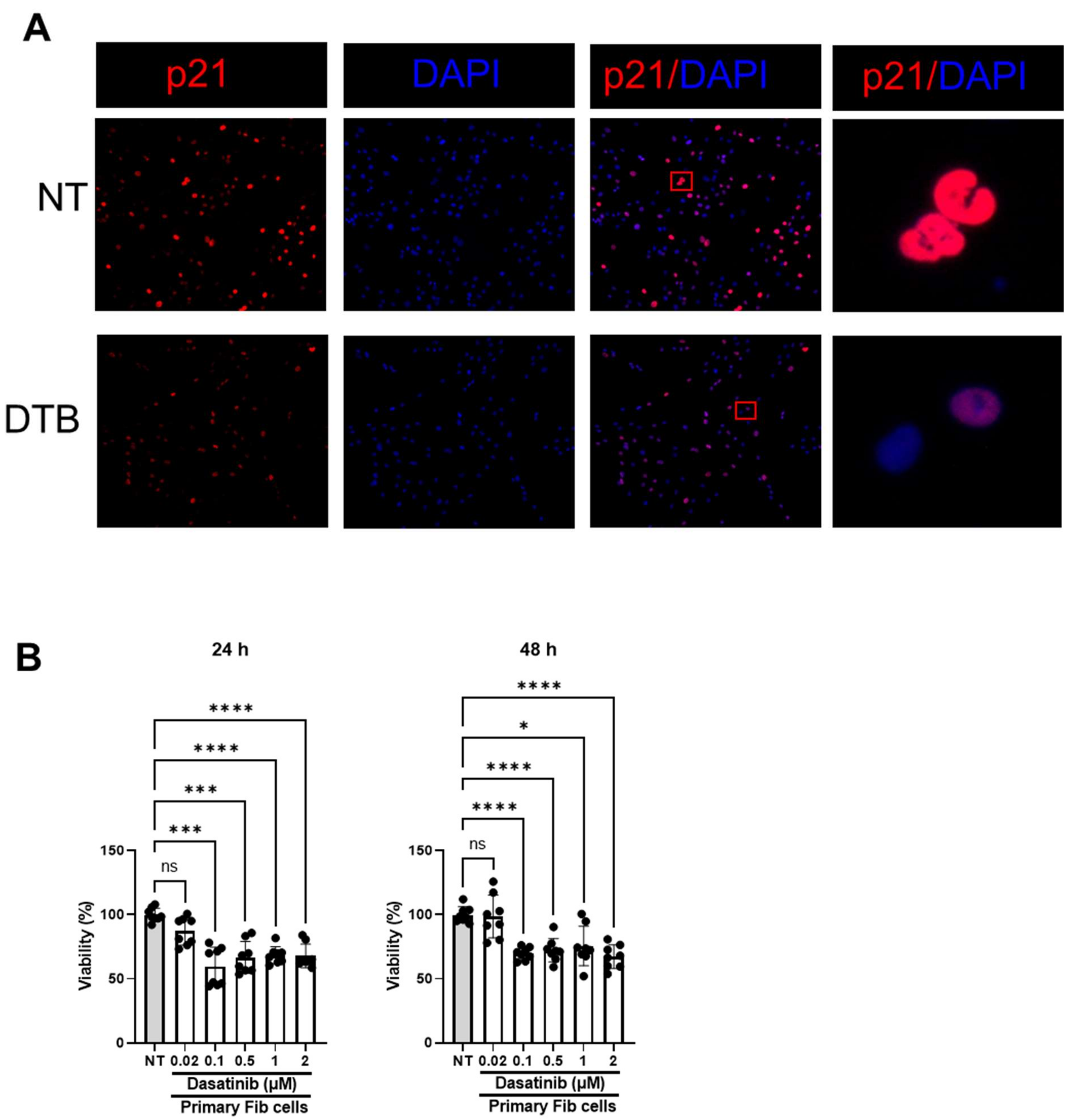

Fig. S4 The expression levels of SOX9 in cultured primary fibroid and myometrial cells. Western blot images (left) and quantification (right) of SOX9 in primary fibroid and myometrial cells (myo=5, fib=5 biological replicates) presented in scatter plots. The α-actinin was used as a loading control to normalize the protein expression data. Shapiro-Wilk test was used to assess whether data are normally distributed or not. Unpaired t test. $*p < 0.05$; $**p < 0.01$; $***p < 0.001$; $****p < 0.0001$.

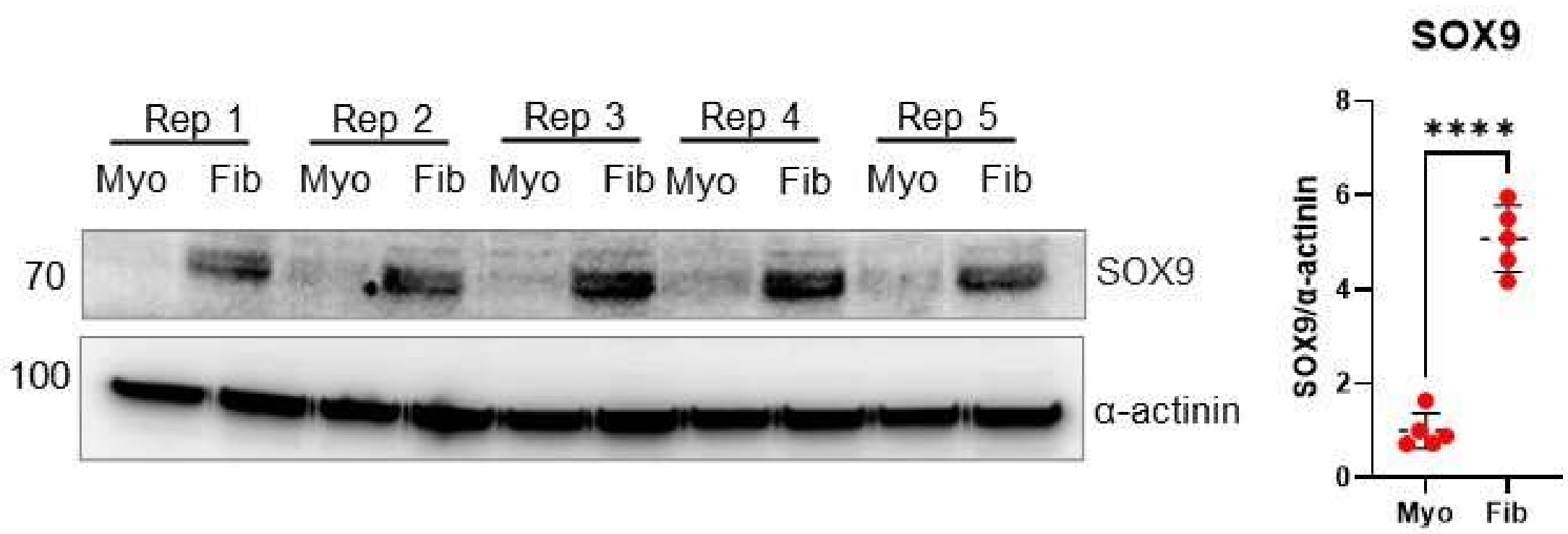

Fig. S5 Effect of ICG-001 on viability of fibroid cells. Viability of fibroid cells was assessed using MTS assay after treatment with ICG-001 at different concentrations (n=8 biological replicates) for 24 and 48 hours. Shapiro-Wilk test was used to assess whether data are normally distributed or not. *$p < 0.05$; **$p < 0.01$; ***$p < 0.001$. One-way ANOVA.

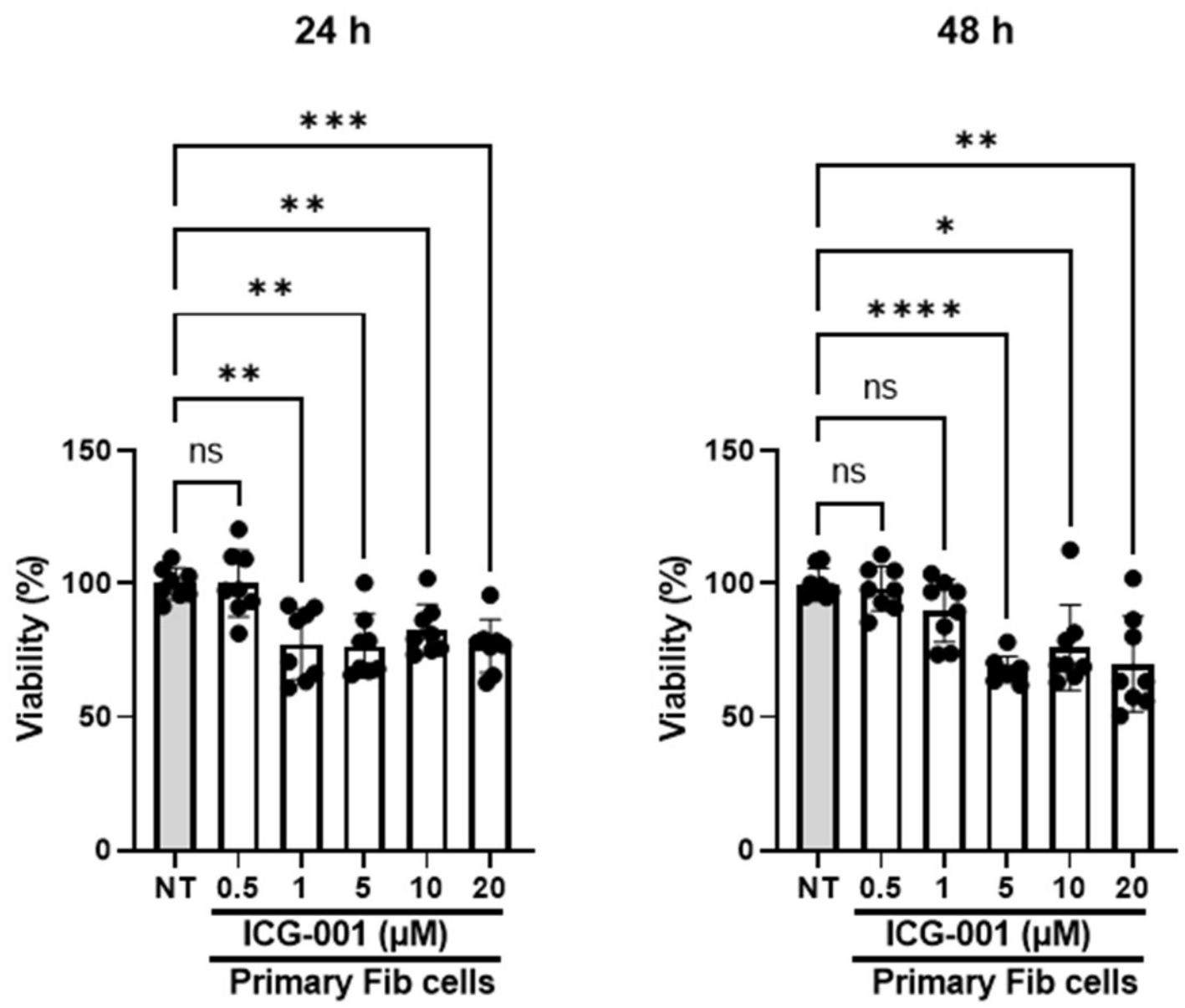

Fig. S6 Effect of PRI-7 on cell viability and p21expression in cultured uterine fibroid cells. A) Chemical structure of PRI-724 and a schematic of its inhibitory action on the β-catenin/CBP complex. **B**) Viability of fibroid cells was assessed using MTS assay after treatment with PRI-7 at different concentrations (n=6 biological replicates) for 24 and 48 hours. **C**) Representative immunofluorescence images (10X) of p21 from four biological replicates in fibroid cells treated with PRI-7 at 1.25 μM for 48 hours. Shapiro-Wilk test was used to assess whether data are normally distributed or not. *$p < 0.05$; **$p < 0.01$; ***$p < 0.001$; ****$p < 0.0001$. One-way ANOVA (**B**).

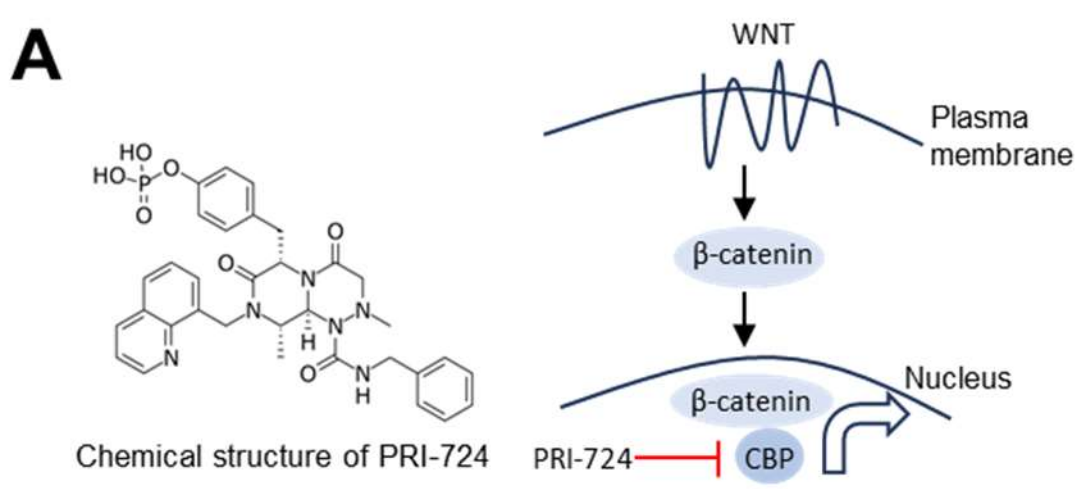

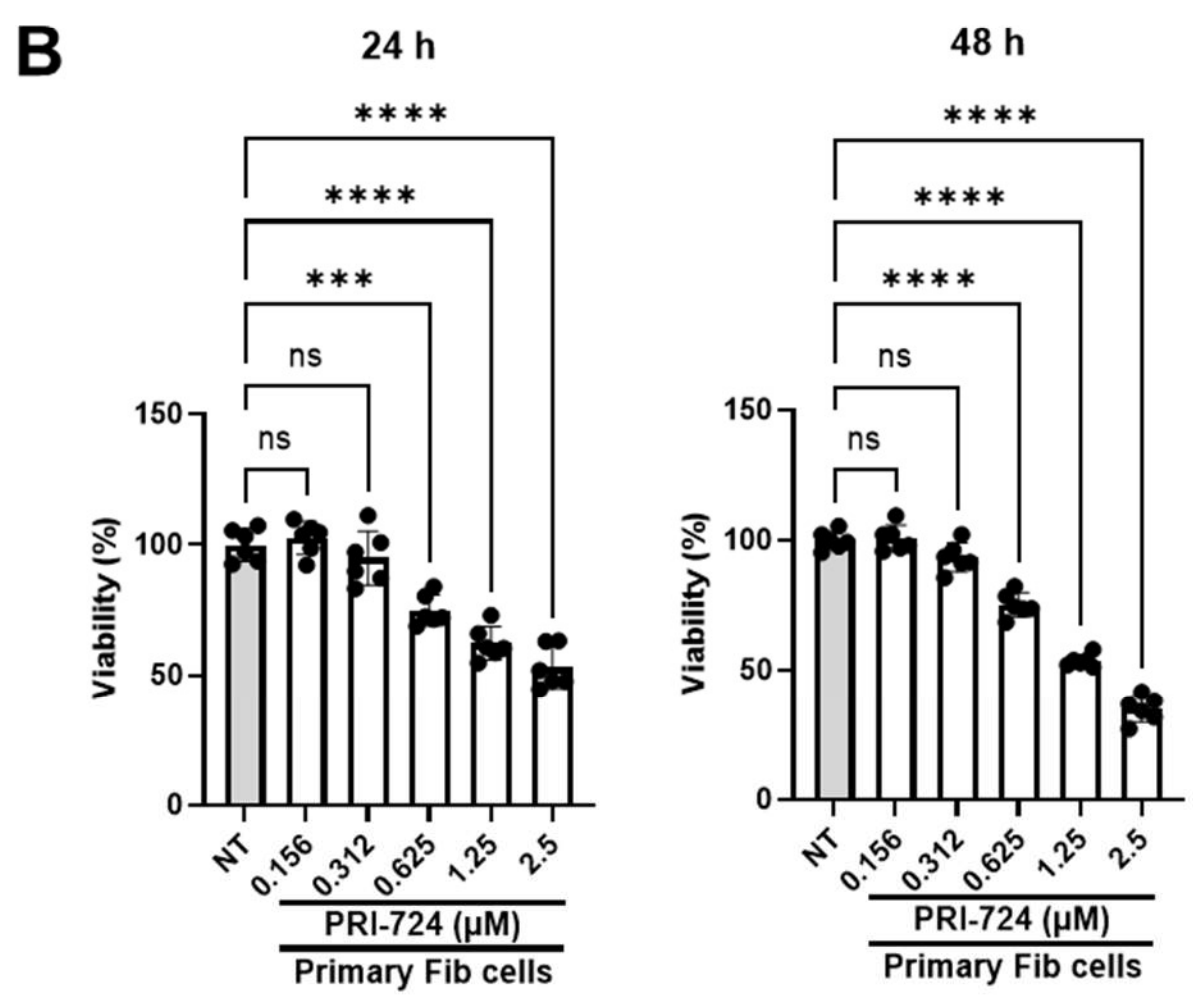

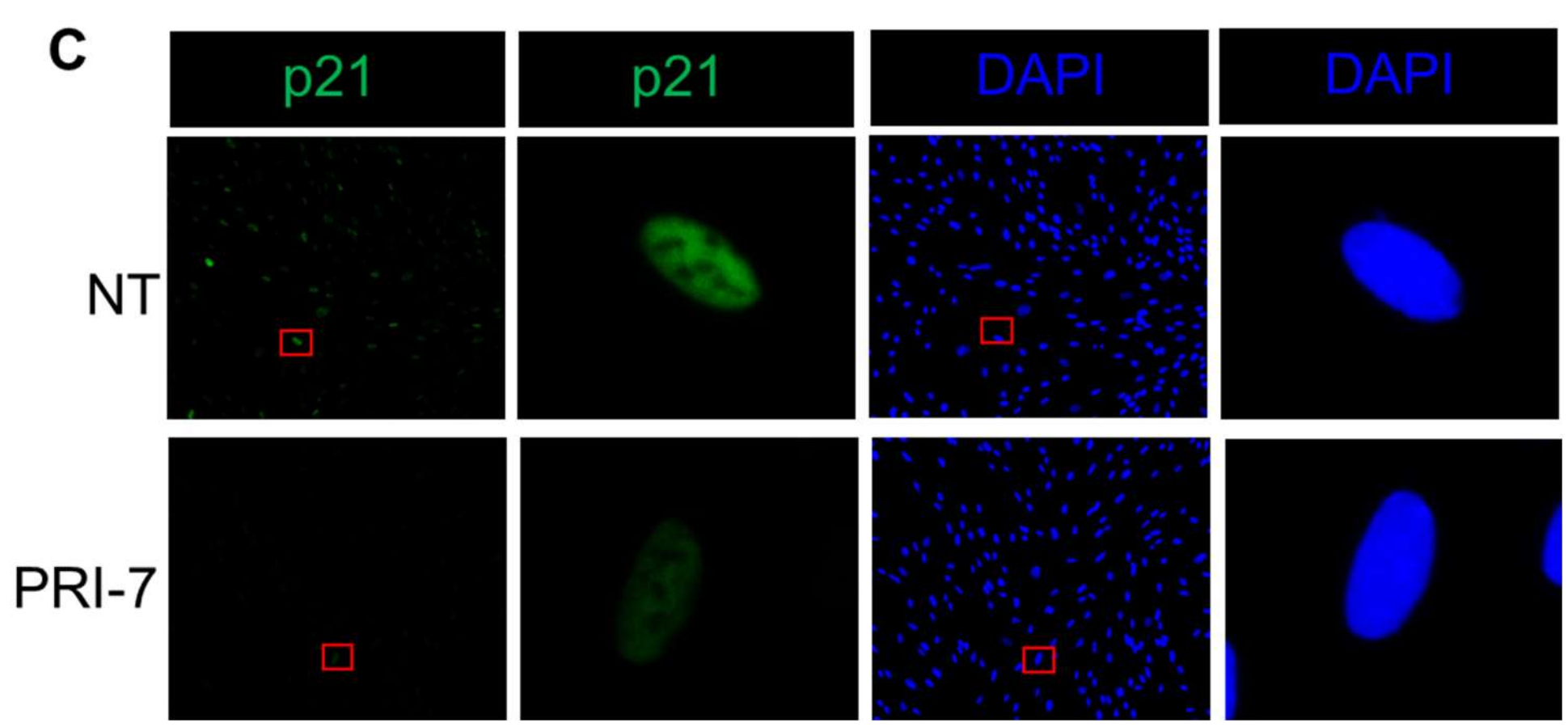

Fig. S7 Effect of PRI-7 on expression of fibronectin (FN1) in cultured uterine fibroid cells. Representative immunofluorescence images (10X) of FN1 from four biological replicates in fibroid cells treated with PRI-7 at 1.25 μM for 48 hours.

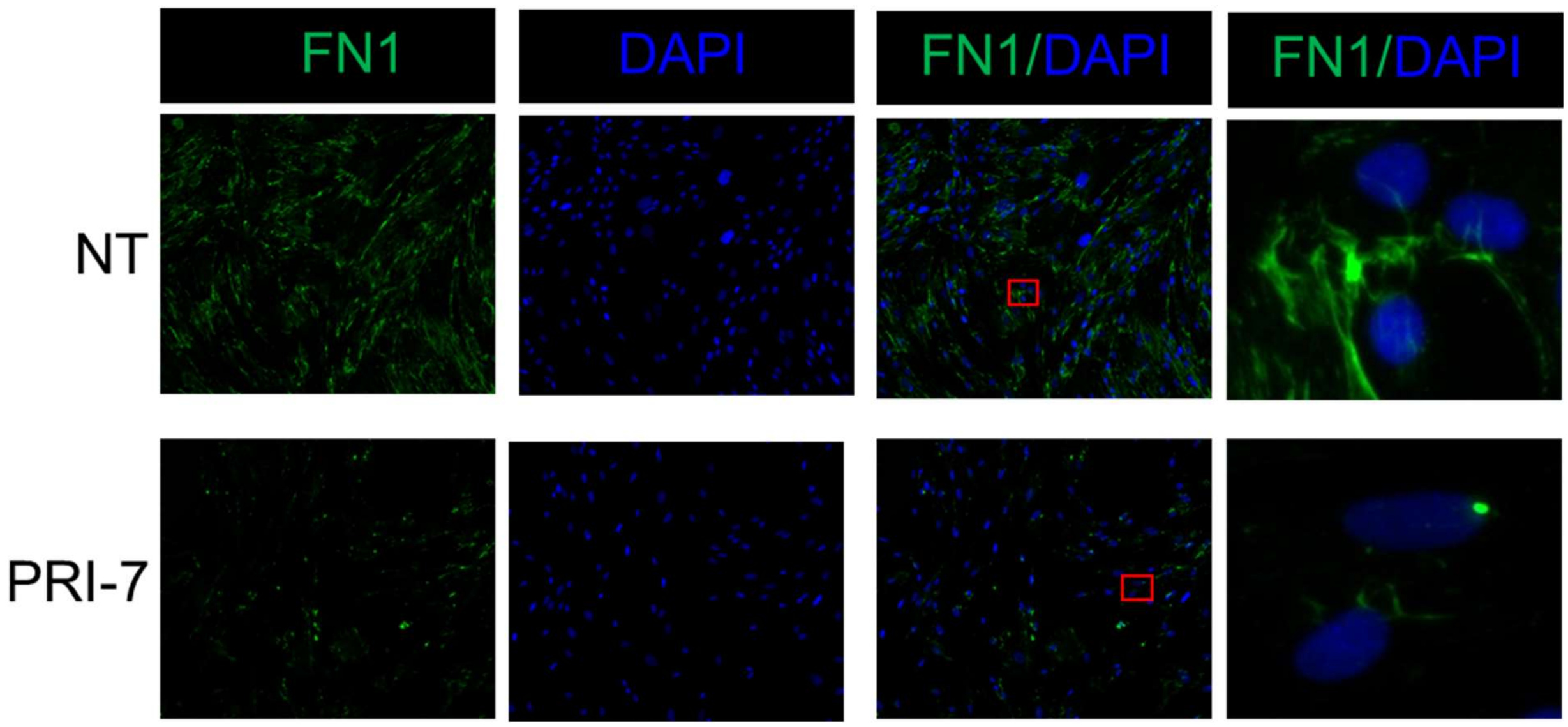

Table S1. List of primary antibodies.

Primary antibodies	Cat no.	Company	Size (kDa)	Host/Isotype	WB dil.	IF
p16INK4a	A11651	Abclonal	16	Rabbit	1: 500	
p16INK4a	10883-1-AP	Proteintech	16	Rabbit	-	1: 100
p21 Waf1/Cip1 (12D1)	2947	Cell Signaling Technology	21	Rabbit/IgG	1: 1000	1: 800
Fibronectin	ab6584	Abcam	238-268	Rabbit	1: 10000	
Fibronectin	26836S	Cell Signaling Technology	300	Rabbit IgG	-	1: 800
PAI-1	11907	Cell Signaling Technology	48	Rabbit/IgG	1: 1000	
α-Smooth Muscle Actin (D4K9N)	19245	Cell Signaling Technology	42	Rabbit/IgG	1: 1000	
Non-phospho (Active) β-Catenin (Ser45) (D2U8Y)	19807	Cell Signaling Technology	92	Rabbit/IgG	1: 1000	
Non-phospho (Active) YAP (Ser127) (E6U8Z)	29495	Cell Signaling Technology	65-78	Rabbit/IgG	1: 500	
Phospho-Smad2 (Ser465/467) (138D4)	3108	Cell Signaling Technology	60	Rabbit/IgG	1: 500	
Cyclin D1	2922	Cell Signaling Technology	36	Rabbit	1: 500	
CTGF	86641	Cell Signaling Technology	35	Rabbit/IgG	1: 500	
CTGF	86641	Cell Signaling Technology	35	Rabbit/IgG	-	1: 50
Sox9 (D8G8H)	82630	Cell Signaling Technology	70	Rabbit/IgG	1: 500	
COL1A1	PA5-29569	Thermo Fisher Scientific	130-250	Rabbit/IgG	1: 500	
COL1A1	39952S	Cell Signaling Technology	220	Rabbit IgG	-	1: 800
Phospho-Src Family (Tyr416)	2101	Cell Signaling Technology	60	Rabbit	1: 500	
LAMC1	92921	Cell Signaling Technology	220-250	Rabbit	1: 1000	
MMP-2 (D4M2N)	40994	Cell Signaling Technology	64,72	Rabbit/IgG	1: 1000	
TIMP1 (D10E6)	8946	Cell Signaling Technology	26	Rabbit/IgG	1: 1000	
α-Actinin (D6F6)	6487	Cell Signaling Technology	100	Rabbit/IgG	1: 2000	

Table S2. List of primers.

Gene name	Direction	Sequence (5'->3')	Direction	Sequence (5'->3')
CDKN2A	Forward	AGGTGGACCTGGAGACTCTCAG	Reverse	TCCTCTTGGAGAAGATCAGCCG
CDKN1A	Forward	CTCGTGCTGATGCTACTGAGGA	Reverse	GGTCGGCGCAGTTGGGCTCC
FN1	Forward	ACAACACCGAGGTGACTGAGAC	Reverse	GGACACAACGATGCTTCCTGAG
PAI1	Forward	GAGAACCTGGGAATGACCGAC	Reverse	TGCCACTCTCGTTCACCTCG
ACTA2	Forward	TCAATGTCCCAGCCATGTAT	Reverse	CAGCACGATGCCAGTTGT
VCAN	Forward	GAAAAGTCAGCCTACCTTGTC	Reverse	GATGCGGAGAAATTCACTGG
TNC	Forward	ATGTCCTCCTGACAGCCGAGAA	Reverse	AGTCACGGTGAGGTTTTCCAGC
LAMC1	Forward	AGATGGAAGCTGAGAATCTGG	Reverse	CGGTCTGCTGTTCAGTCTTG
POSTN	Forward	CAGCAAACCACCTTCACGGATC	Reverse	TTAAGGAGGCGCTGAACCATGC
COL1A1	Forward	GATTCCCTGGACCTAAAGGTGC	Reverse	AGCCTCTCCATCTTTGCCAGCA
COL3A1	Forward	TGGTCTGCAAGGAATGCCTGGA	Reverse	TCTTTCCCTGGGACACCATCAG
COL4A2	Forward	GGATAACAGGCGTGACTGGAGT	Reverse	CTTTGCCACCAGGCAGTCCAAT
COL5A1	Forward	GGAGATGATGGTCCCAAAGGCA	Reverse	CCATCATCTCCTTTGTCACCAGG
COL6A1	Forward	GCCTTCCTGAAGAATGTCACCG	Reverse	TCCAGCAGGATGGTGATGTCAG
LOX1	Forward	GAAACCCTTGCTCGGAAGCTGA	Reverse	CAGATCCAGTCTTGCGGACAAG
P4HA1	Forward	GCCAAAGCTCTGTTACGTCTCC	Reverse	CAAAGCAGTCCTCAGCCGTTAG
P4HA2	Forward	CCACTGATGAGGACGAGATAGG	Reverse	GTCATCCACACTCAGCATTGCC
GLB1	Forward	CACTCCACAATCAAGACCGAAGC	Reverse	CTGTGCTGCATAGGGTGAGTTG
TGFB1	Forward	TACCTGAACCCGTGTTGCTCTC	Reverse	GTTGCTGAGGTATCGCCAGGAA
IGF1	Forward	CTCTTCAGTTCGTGTGTGGAGAC	Reverse	CAGCCTCCTTAGATCACAGCTC
ARF1	Forward	GCCACTACTTCCAGAACACACA	Reverse	TCGTTCACACGCTCTCTGTC
