# Supplementary material for: Src and β-catenin control a senescence-fibrotic phenotype in uterine fibroid cells
Source: Sci Rep. 2026 Jun 26;16:29418. doi: 10.1038/s41598-026-57742-5 (PMC13594328; doi:10.1038/s41598-026-57742-5)
Supplement: Supplementary file 2 [file 41598_2026_57742_MOESM2_ESM.pdf]

Supplementary Info File 2

Fig. S1. Full immunoblots related to Fig. 1A, D-E. A) The expression levels of p16 in primary myometrial and fibroid cells isolated from patient-matched tissues (myo=9, fib=9 from 9 patients). **D**) The expression levels of p16 in primary fibroid cells cultured on a soft (930 kPa) and a stiff substrate (3 GPa) for 48 hours (soft=5, stiff= 5 fibroids from 5 patients). **E**) The expression levels of p21 in primary fibroid cells cultured on a soft (930 kPa) and a stiff substrate (3 GPa) for 48 hours (soft=5, stiff= 5 fibroids from 5 patients). Membranes were cut into several pieces (based on the molecular weight of proteins of interest) prior to hybridization with primary antibodies (p16, p21, and α-actinin) during western blot. MW (in kDa) are indicated. The α-actinin was used as loading control to normalize the data.

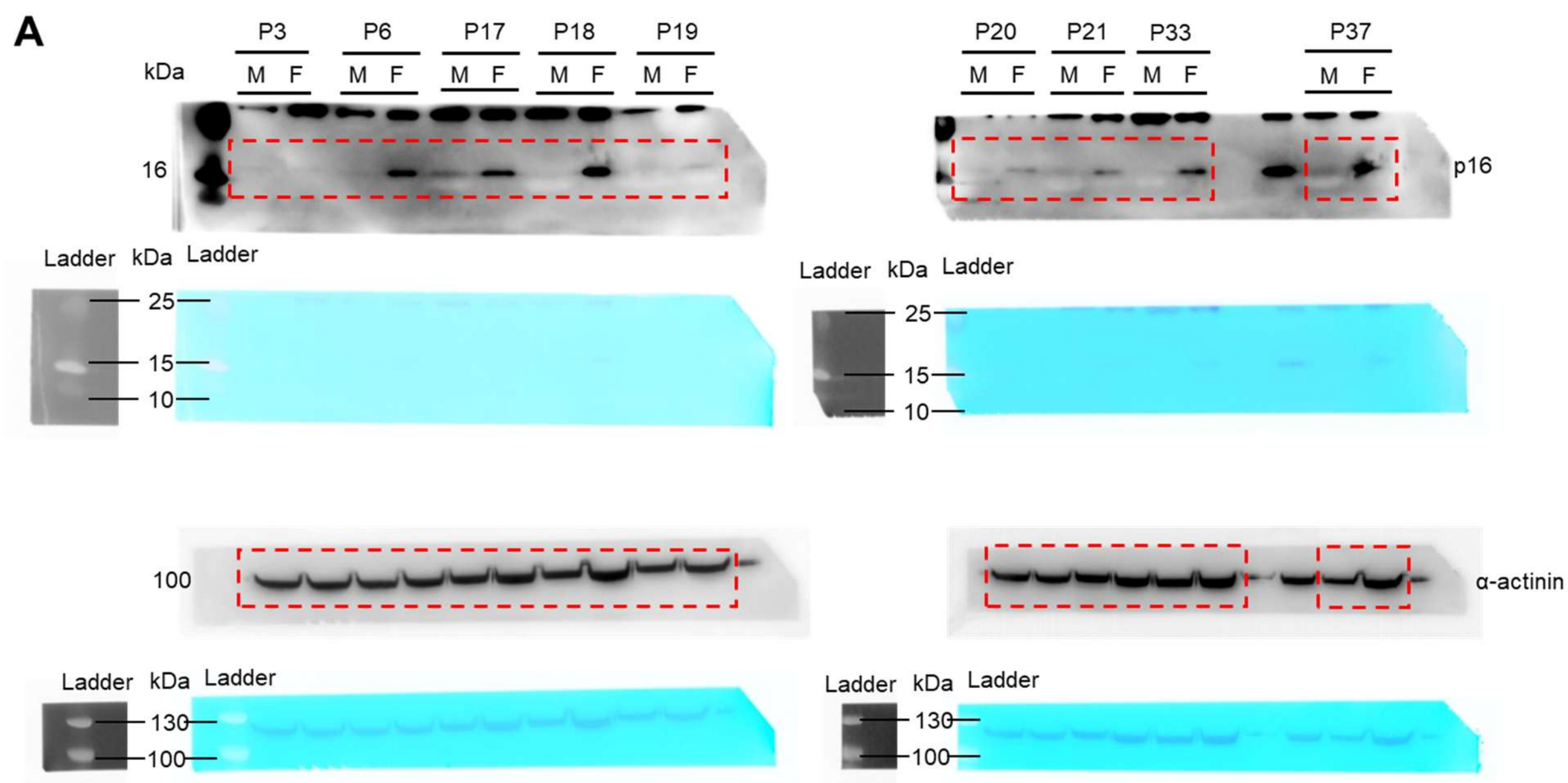

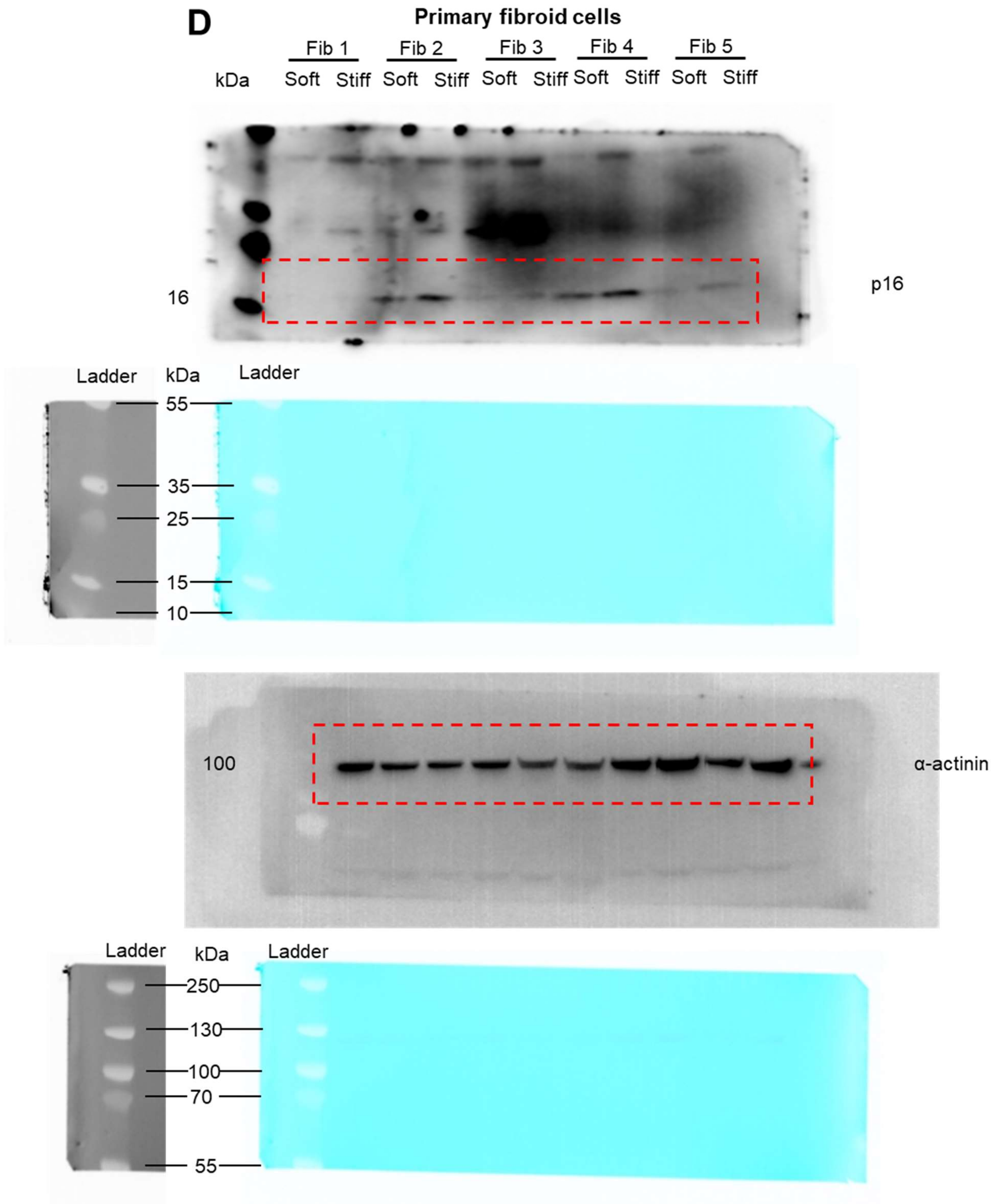
D
Primary fibroid cells
Fib 1
Fib 2
Fib 3
Fib 4
Fib 5
kDa
Soft Stiff Soft Stiff Soft Stiff Soft Stiff Soft Stiff
16
p16
Ladder
kDa
Ladder
55
35
25
15
10
100
α-actinin
Ladder
kDa
Ladder
250
130
100
70
55

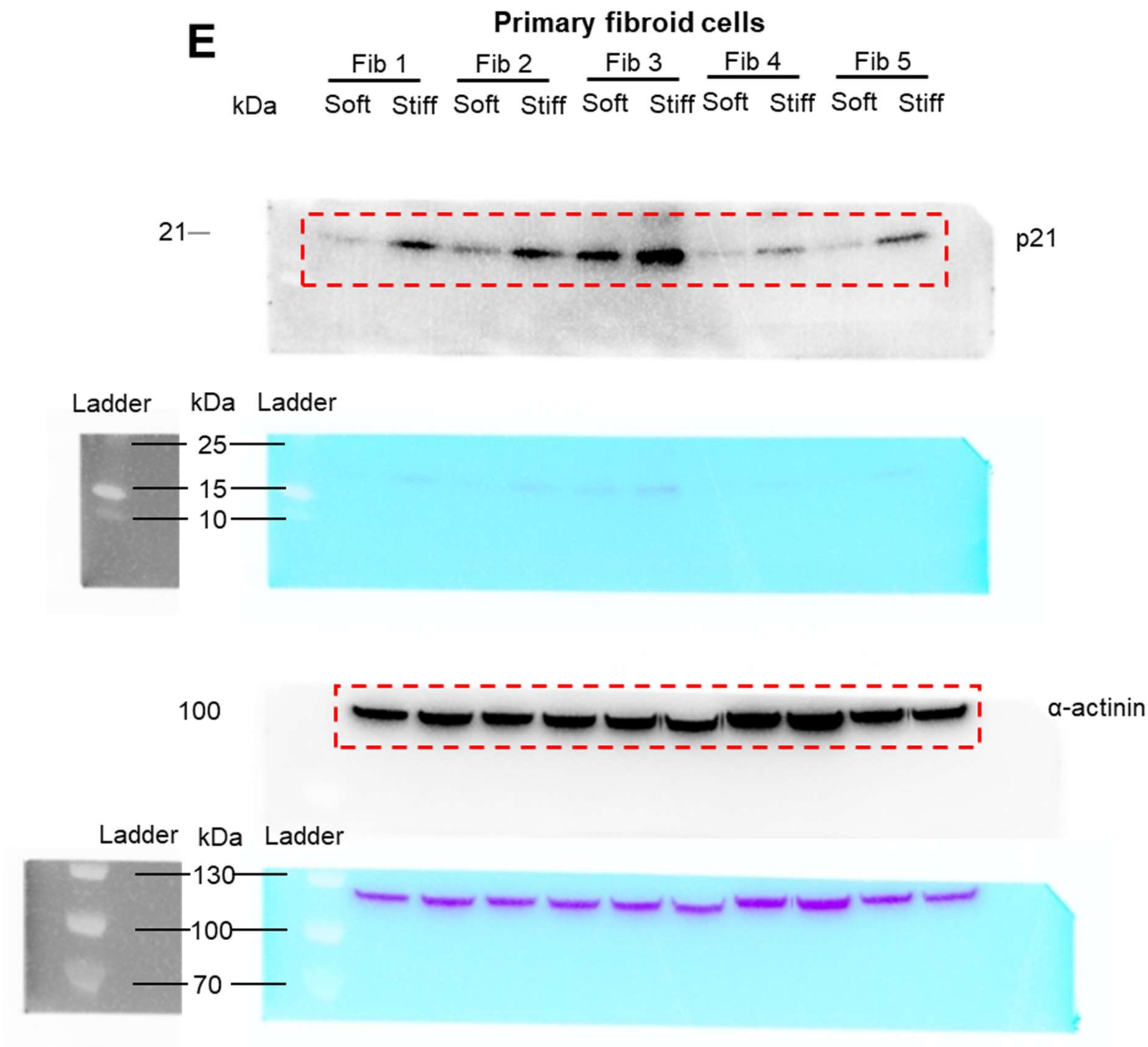
E
Primary fibroid cells
Fib 1
Fib 2
Fib 3
Fib 4
Fib 5
kDa
Soft Stiff Soft Stiff Soft Stiff Soft Stiff Soft Stiff
21
p21
Ladder
kDa
Ladder
25
15
10
100
α-actinin
Ladder
kDa
Ladder
130
100
70

Fig. S2. Full immunoblots related to Fig. 2A. A) The expression levels of fibronectin (FN1), PAI1, and α-SMA in primary fibroid cells cultured on a soft (930 kPa) and a stiff substrate (3 GPa) for 48 hours (soft=5, stiff=5 from independent cultures). Membranes were cut into several pieces (based on the molecular weight of proteins of interest) prior to hybridization with primary antibodies (FN1, PAI1, α-SMA, and α-actinin) during western blot. MW (in kDa) are indicated. The α-actinin was used as loading control to normalize the data.

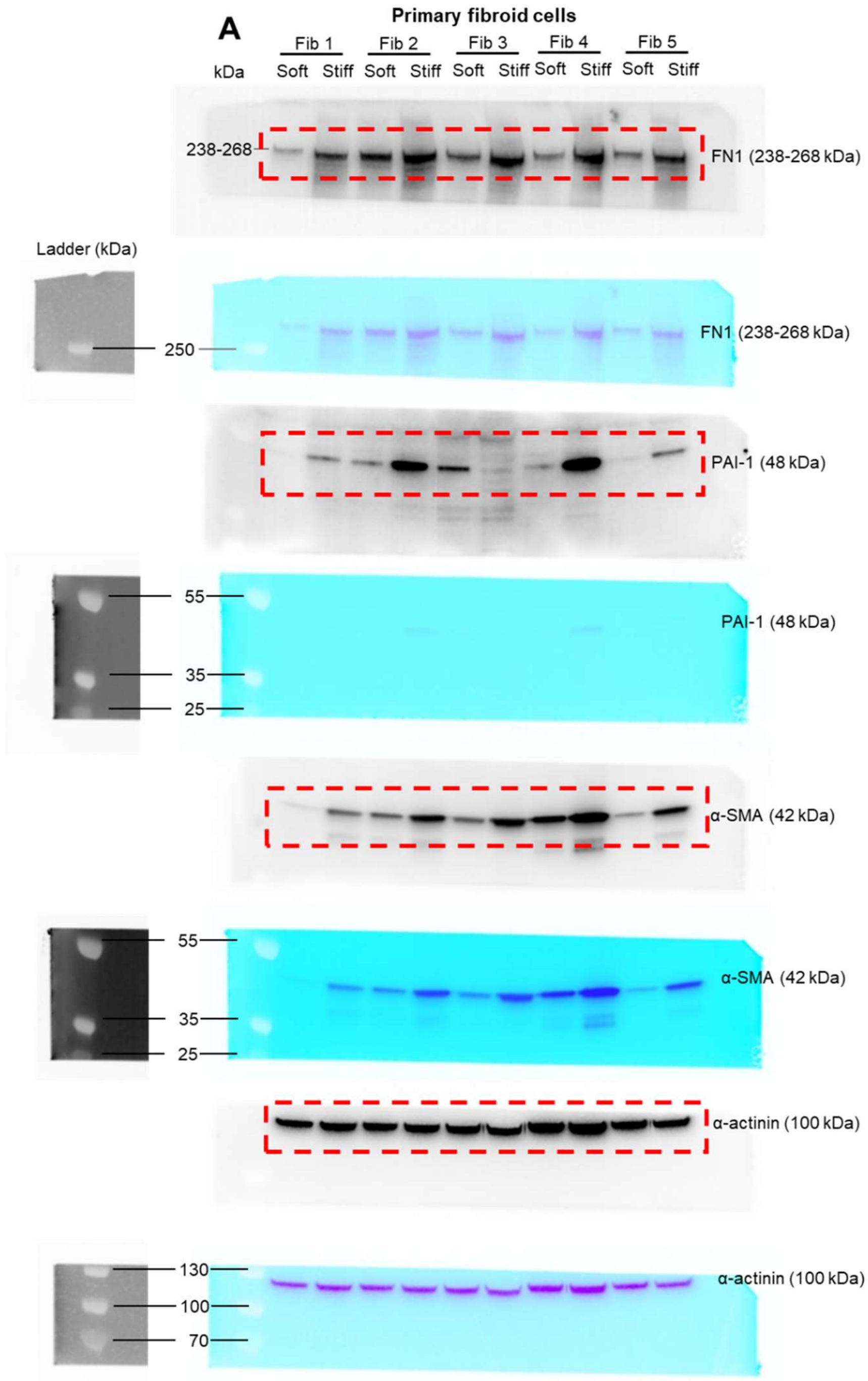

Fig. S3. Full immunoblots related to Fig. 3A-B. A) The expression levels of β-catenin (active), non-p-YAP (active), and phospho-Smad2 in primary fibroid cells cultured on a soft (930 kPa) and a stiff substrate (3 GPa) for 48 hours (soft=5, stiff=5 from independent cultures). **B**) The expression levels of cyclin D1 and CTGF in primary fibroid cells cultured on a soft (930 kPa) and a stiff substrate (3 GPa) for 48 hours (soft=5, stiff=5 from independent cultures). Membranes were cut into several pieces (based on the molecular weight of proteins of interest) prior to hybridization with primary antibodies (β-catenin, non-p-YAP, and phospho-Smad2, cyclin D1, CTGF, and α-actinin)] during western blot. MW (in kDa) are indicated. The α-actinin was used as loading control to normalize the data.

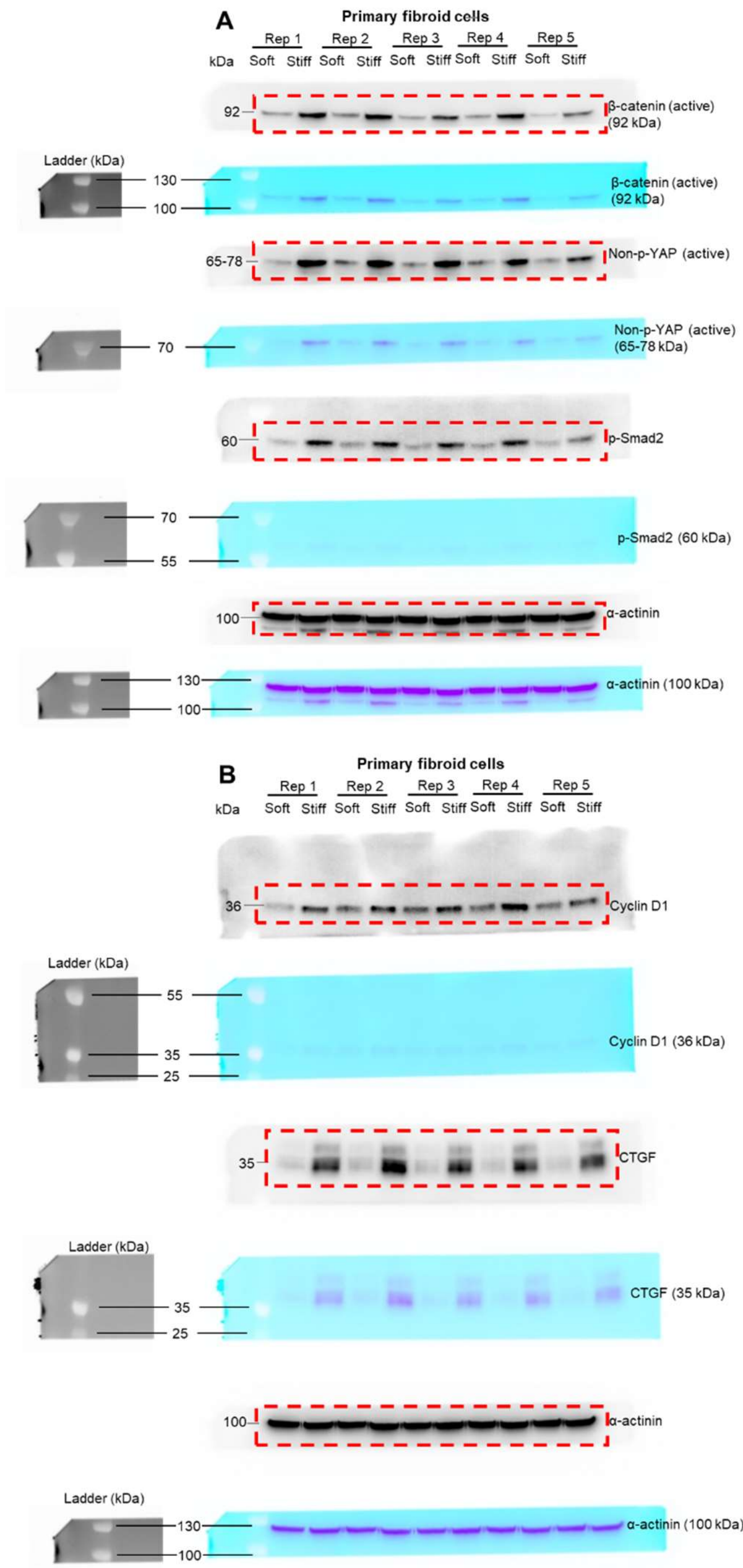

Fig. S4. Full immunoblots related to Fig. 4C-D. C) The expression levels of phospho-Src in primary fibroid cells cultured on a stiff substrate (3 GPa) for 48 hours (control=6, dasatinib=6 replicates from independent cultures). **D**) The expression levels of p16 and p21 in primary fibroid cells cultured on a stiff substrate (3 GPa) for 48 hours (control=6, dasatinib=6 replicates from independent cultures). Membranes were cut into several pieces (based on the molecular weight of proteins of interest) prior to hybridization with primary antibodies (phospho-Src, p16, p21, and α-actinin) during western blot. MW (in kDa) are indicated. The α-actinin was used as a loading control to normalize the protein expression data.

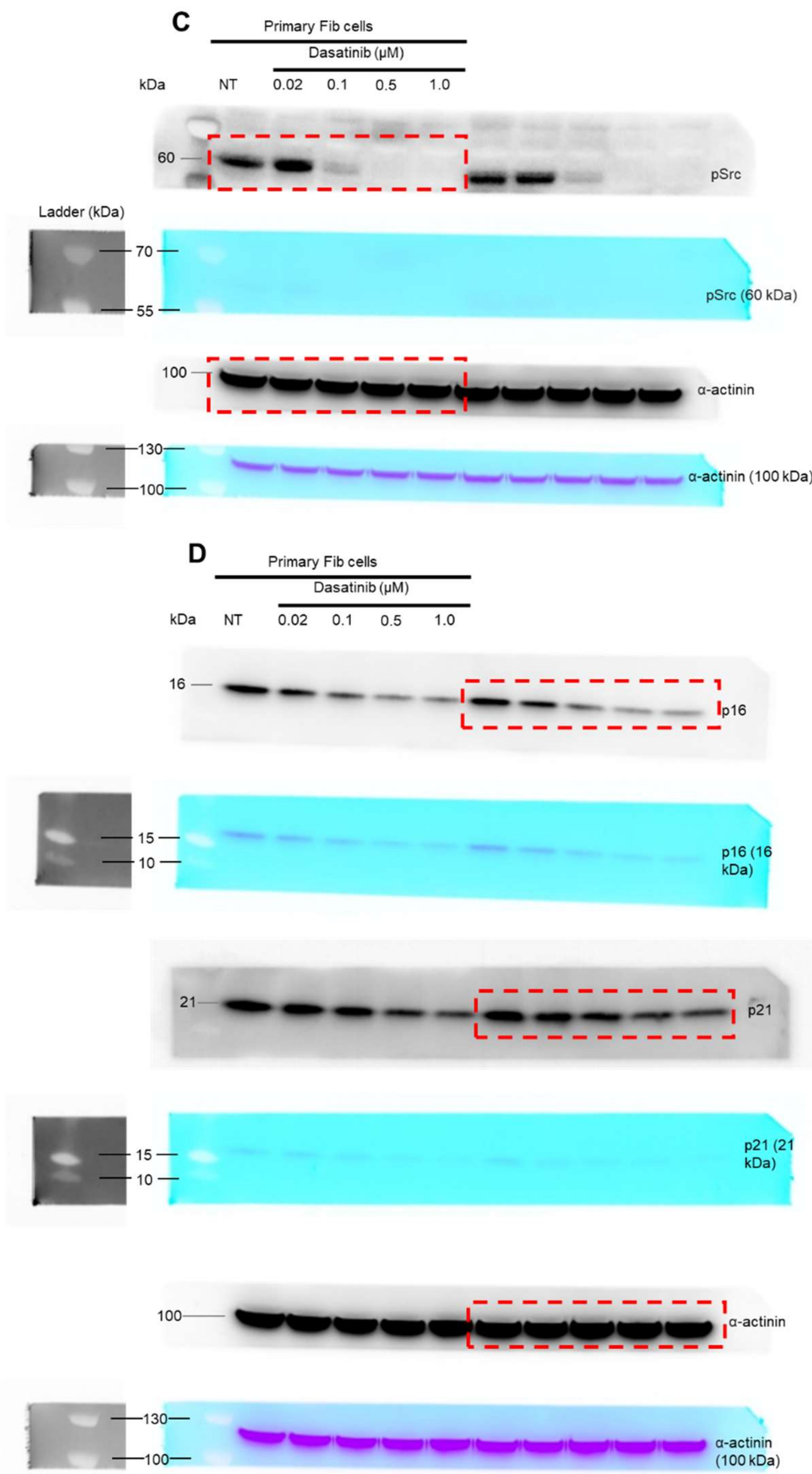

Fig. S5. Full immunoblots related to Fig. 5. The expression levels of fibronectin (FN1), LAMC1, COL1A1, PAI-1, MMP2, and TIMP1 in primary fibroid cells cultured a stiff substrate (3 GPa) for 48 hours (control=6, dasatinib=6 replicates from independent cultures). Membranes were cut into several pieces (based on the molecular weight of proteins of interest) prior to hybridization with primary antibodies (FN1, LAMC1, COL1A1, PAI-1, MMP2, and TIMP1, and α-actinin) during western blot. MW (in kDa) are indicated. The α-actinin was used as a loading control to normalize the protein expression data.

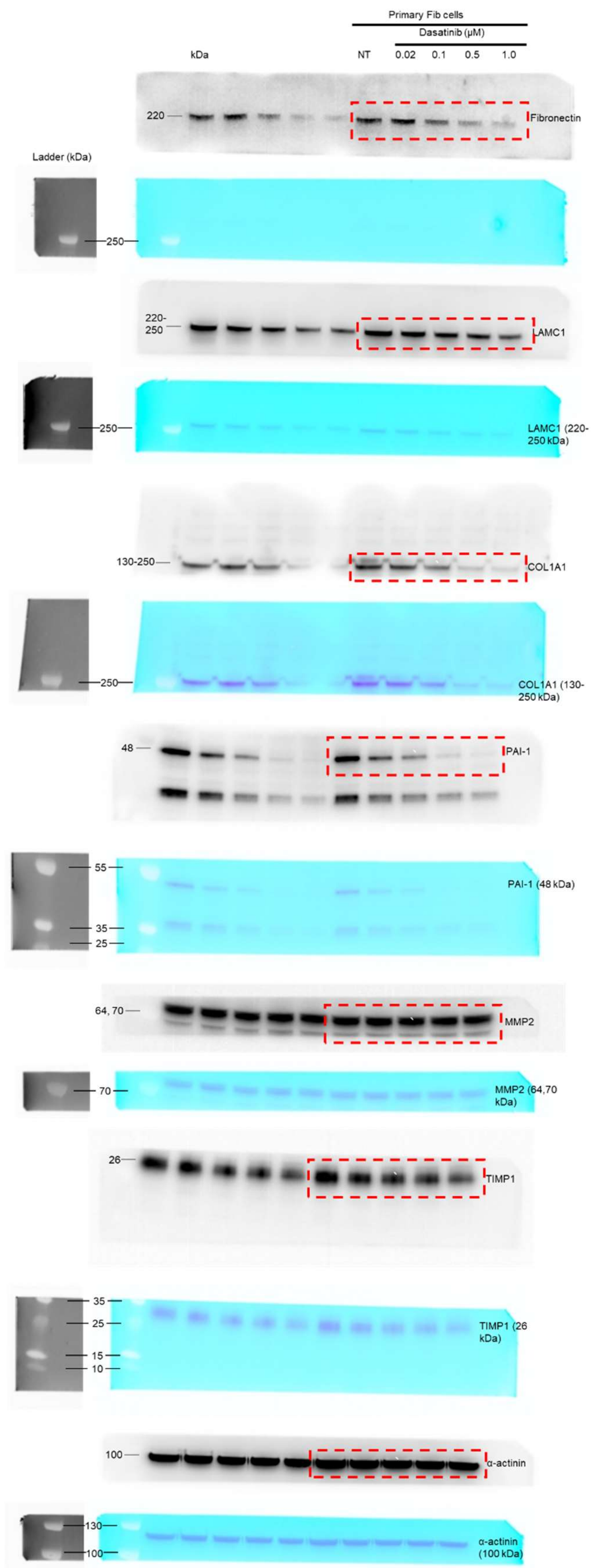

Primary Fib cells
Dasatinib (μM)
kDa
NT
0.02
0.1
0.5
1.0
220
Fibronectin
Ladder (kDa)
250
220-250
LAMC1
250
LAMC1 (220-250 kDa)
130-250
COL1A1
250
COL1A1 (130-250 kDa)
48
PAI-1
55
35
25
PAI-1 (48 kDa)
64, 70
MMP2
70
MMP2 (64,70 kDa)
26
TIMP1
35
25
15
10
TIMP1 (26 kDa)
100
α-actinin
130
100
α-actinin (100 kDa)

Fig. S6. Full immunoblots related to Fig. 6A-B. A) The expression levels of β-catenin (active) in primary fibroid cells cultured a stiff substrate (3 GPa) for 48 hours (control=6, dasatinib=6 replicates from independent cultures). **B**) The expression levels of cyclin D1, CTGF, and SOX9 in primary fibroid cells cultured a stiff substrate (3 GPa) for 48 hours (control=6, dasatinib=6 replicates from independent cultures). Membranes were cut into several pieces (based on the molecular weight of proteins of interest) prior to hybridization with primary antibodies (β-catenin, cyclin D1, CTGF, SOX9, and α-actinin) during western blot. MW (in kDa) are indicated. The α-actinin was used as a loading control to normalize the protein expression data.

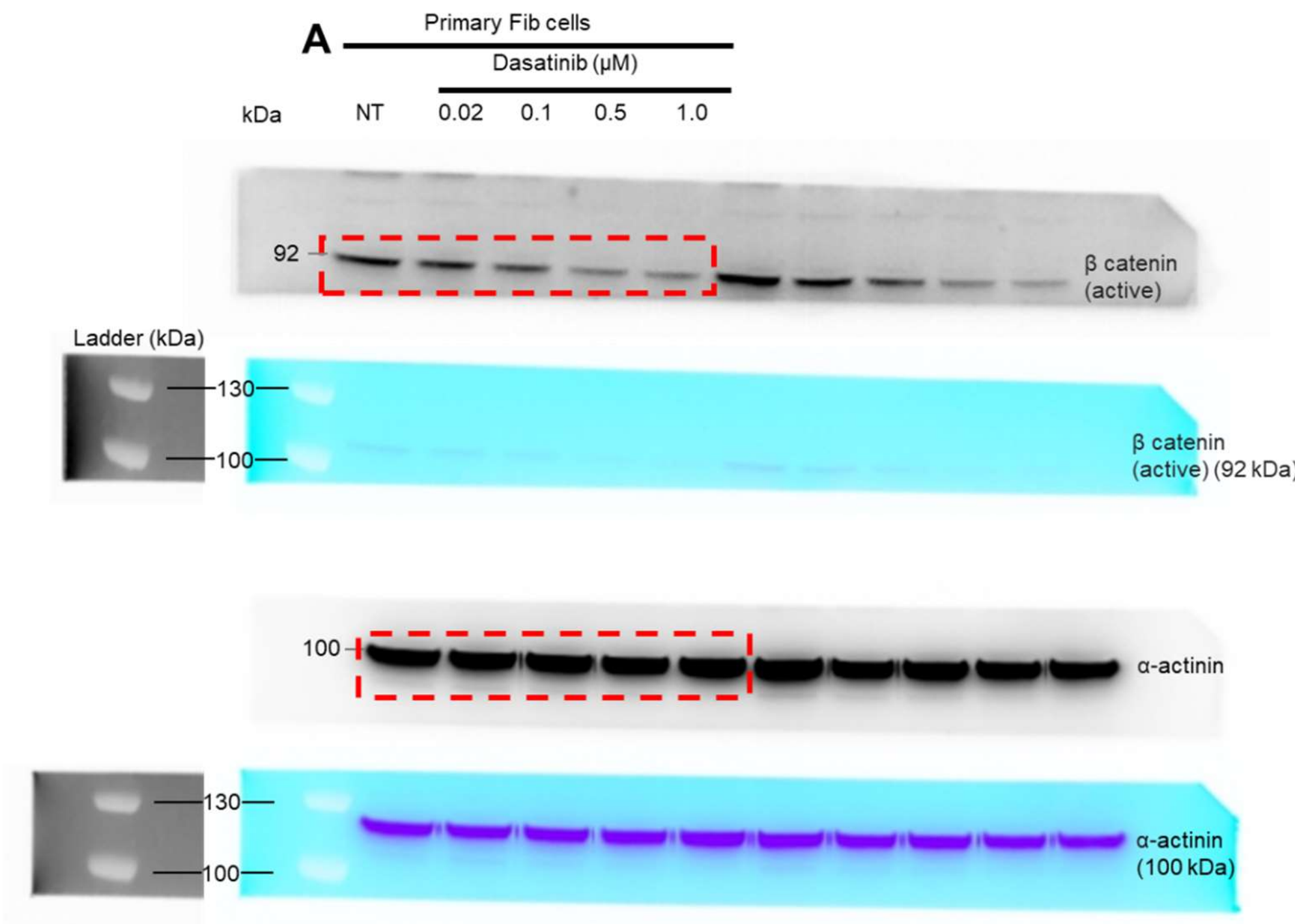

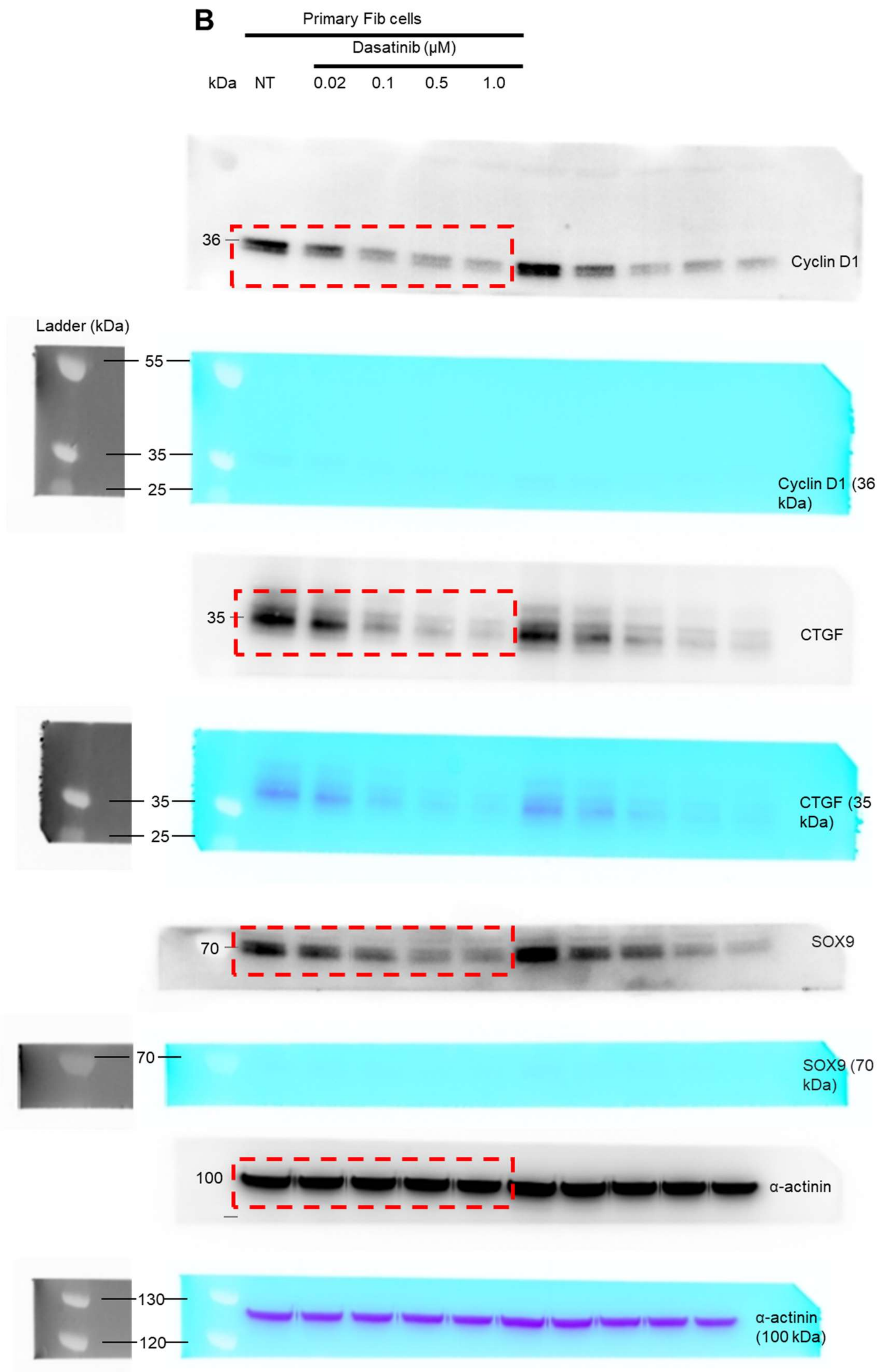
B
Primary Fib cells
Dasatinib (μM)
kDa
NT
0.02
0.1
0.5
1.0
36
Cyclin D1
Ladder (kDa)
55
35
25
Cyclin D1 (36 kDa)
35
CTGF
35
25
CTGF (35 kDa)
70
SOX9
70
SOX9 (70 kDa)
100
α-actinin
130
120
α-actinin (100 kDa)

Fig. S7. Full immunoblots related to Fig. 7B-E. B) The expression levels of β-catenin (active) in primary fibroid cells cultured a stiff substrate (3 GPa) for 48 hours (control=4, ICG-001=4 replicates from independent cultures). **C**) The expression levels of cyclin D1, CTGF, and SOX9 in primary fibroid cells cultured a stiff substrate (3 GPa) for 48 hours (control=4, ICG-001=4 replicates from independent cultures). **D**) The expression levels of p16, and p21 in primary fibroid cells cultured on a stiff substrate (3 GPa) for 48 hours (control=4, ICG-001=4 replicates from independent cultures). **E**) The expression levels of FN1, COL1A1, α-SMA, and PAI-1 in primary fibroid cells cultured a stiff substrate (3 GPa) for 48 hours (control=4, ICG-001=4 replicates from independent cultures). Membranes were cut into several pieces (based on the molecular weight of proteins of interest) prior to hybridization with primary antibodies (β-catenin, cyclin D1, CTGF, SOX9, p16, p21, FN1, COL1A1, PAI-1, α-SMA and α-actinin) during western blot. MW (in kDa) are indicated. The α-actinin was used as loading control to normalize the data.

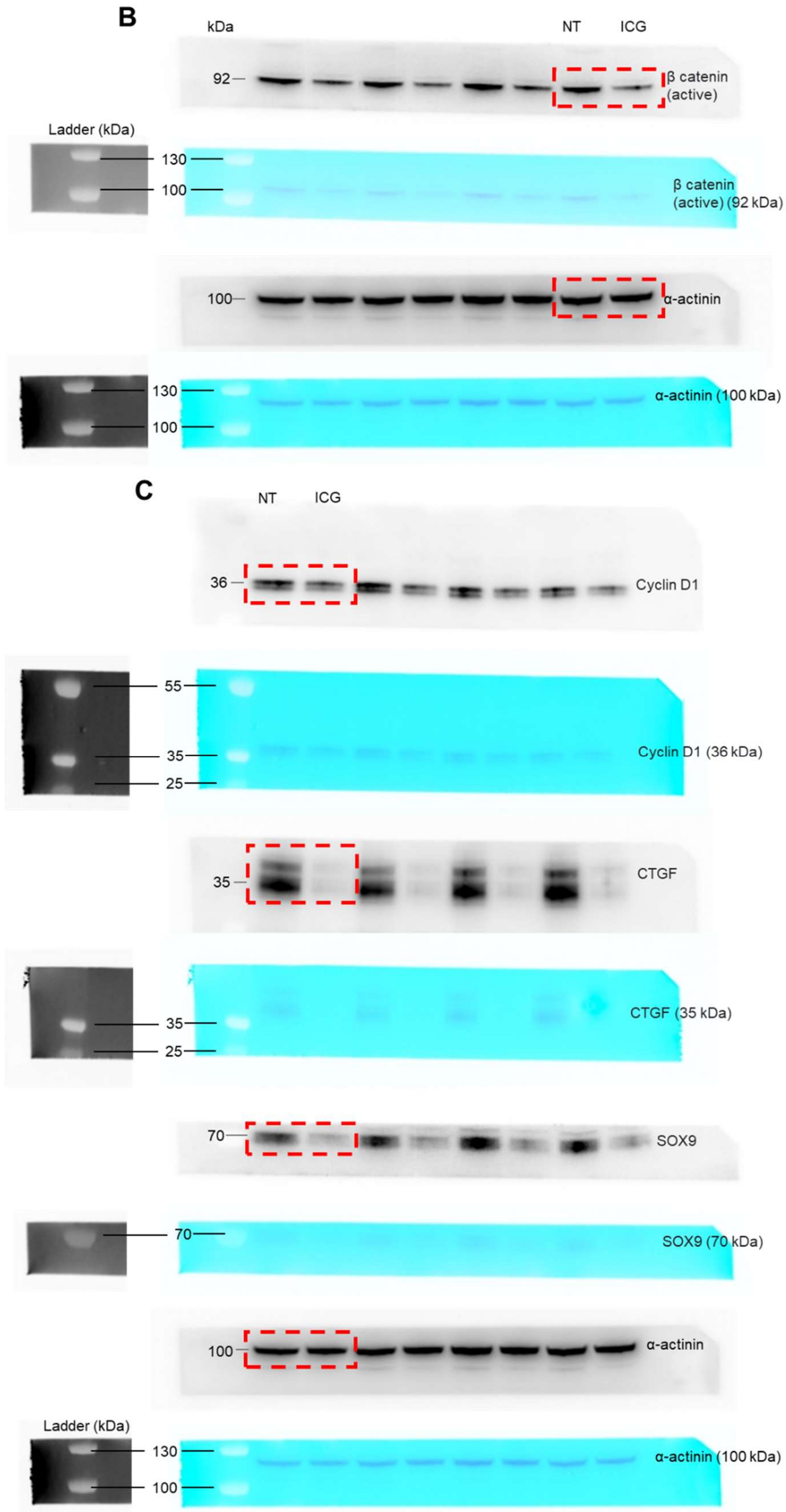

B
kDa
NT
ICG
92
β catenin (active)
Ladder (kDa)
130
100
β catenin (active) (92 kDa)
100
α-actinin
130
100
α-actinin (100 kDa)
C
NT
ICG
36
Cyclin D1
55
35
25
Cyclin D1 (36 kDa)
35
CTGF
35
25
CTGF (35 kDa)
70
SOX9
70
SOX9 (70 kDa)
100
α-actinin
Ladder (kDa)
130
100
α-actinin (100 kDa)

D

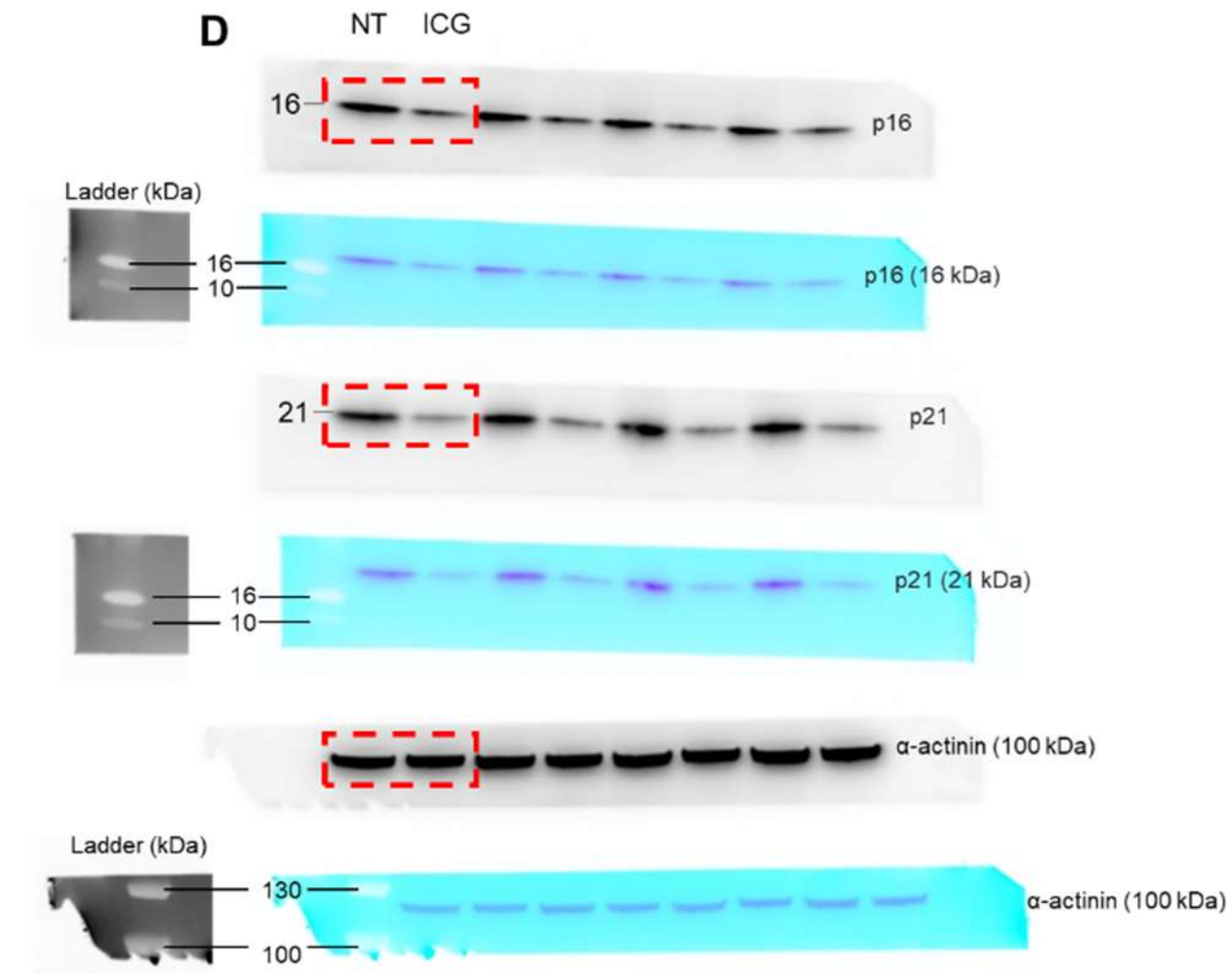

NT
ICG
16
p16
Ladder (kDa)
16
10
p16 (16 kDa)
21
p21
16
10
p21 (21 kDa)
α-actinin (100 kDa)
Ladder (kDa)
130
100
α-actinin (100 kDa)

E

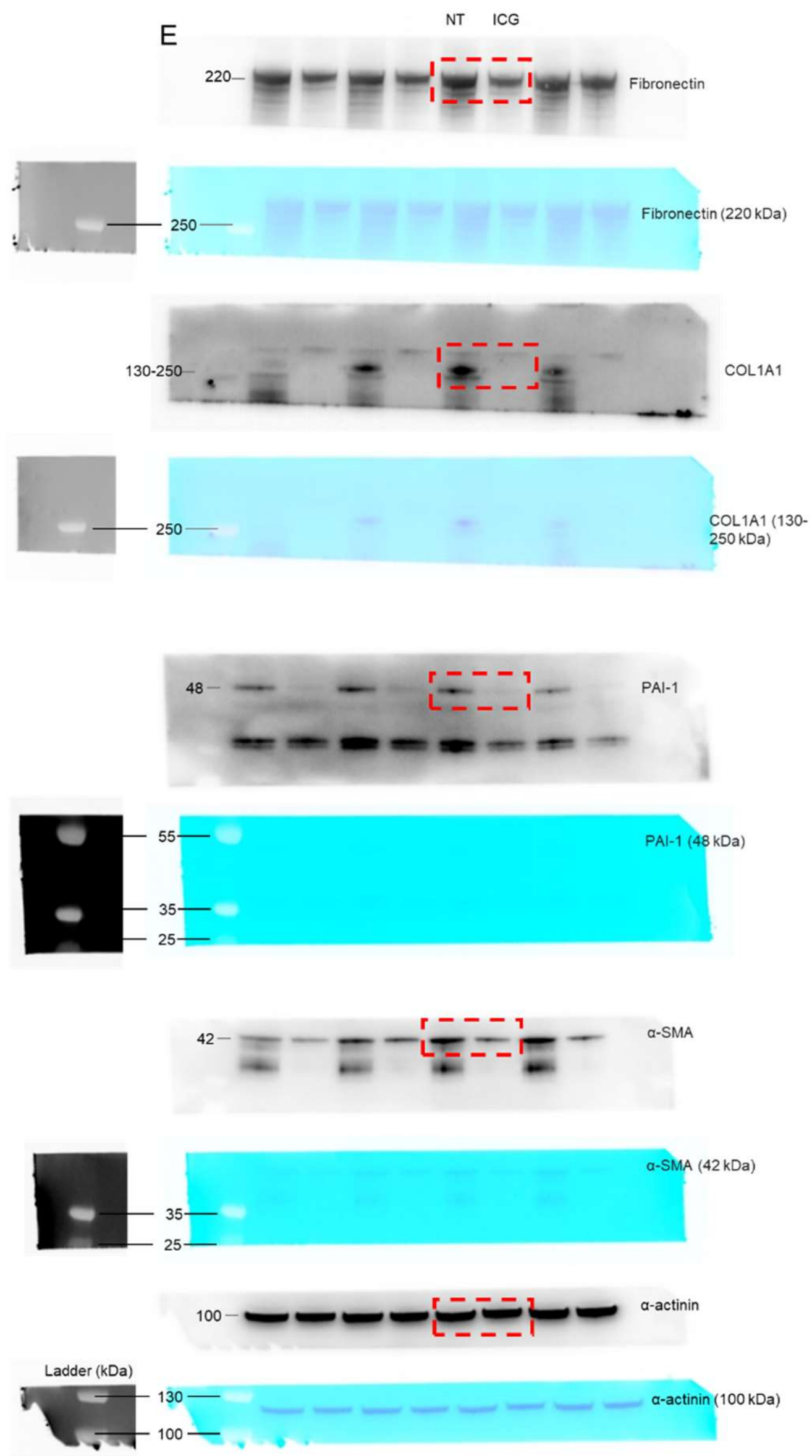

NT
ICG
220
Fibronectin
250
Fibronectin (220 kDa)
130-250
COL1A1
250
COL1A1 (130-250 kDa)
48
PAI-1
55
35
25
PAI-1 (48 kDa)
42
α-SMA
35
25
α-SMA (42 kDa)
100
α-actinin
Ladder (kDa)
130
100
α-actinin (100 kDa)

Fig. S8. Full immunoblots related to Fig. 8A, C. A) The expression levels of β-catenin (active) and CTGF in primary fibroid cells cultured on a stiff substrate (3 GPa) for 48 hours (control=8, PRI-7=8 replicates from independent cultures). **C**) The expression levels of p16 and p21 in primary fibroid cells cultured a stiff substrate (3 GPa) for 48 hours (control=8, PRI-7=8 replicates from independent cultures). Membranes were cut into several pieces (based on the molecular weight of proteins of interest) prior to hybridization with primary antibodies (β-catenin, CTGF, p16, p21, and α-actinin) during western blot. MW (in kDa) are indicated. The α-actinin was used as a loading control to normalize the protein expression data.

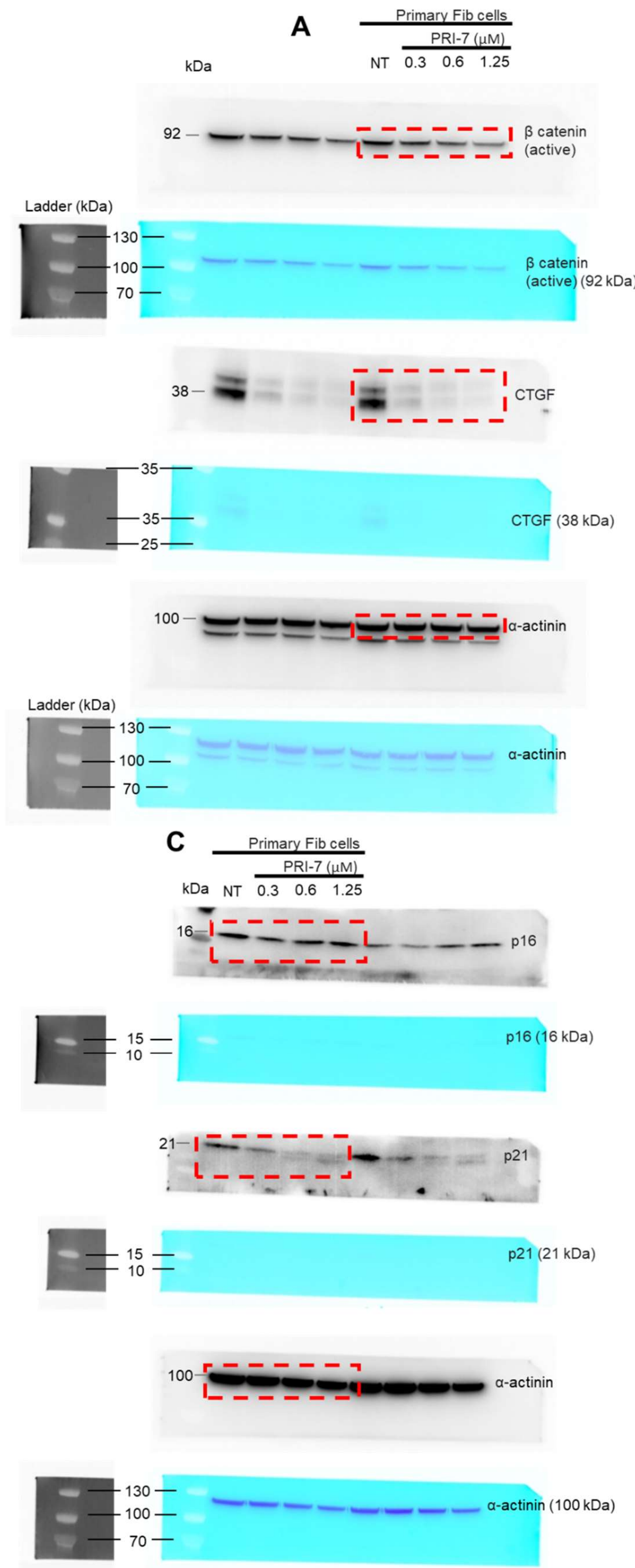

A
Primary Fib cells
PRI-7 (μM)
kDa
NT 0.3 0.6 1.25
92
β catenin (active)
Ladder (kDa)
130
100
70
β catenin (active) (92 kDa)
38
CTGF
35
35
25
CTGF (38 kDa)
100
α-actinin
Ladder (kDa)
130
100
70
α-actinin
C
Primary Fib cells
PRI-7 (μM)
kDa
NT 0.3 0.6 1.25
16
p16
15
10
p16 (16 kDa)
21
p21
15
10
p21 (21 kDa)
100
α-actinin
130
100
70
α-actinin (100 kDa)

Fig. S9. **Full immunoblots related to Fig. 9A. A**) The expression levels of fibronectin, LAMC1, PAI-1 in primary fibroid cells cultured on a stiff substrate (3 GPa) for 48 hours (control=8, PRI-7=8 replicates from independent cultures). Membranes were cut into several pieces (based on the molecular weight of proteins of interest) prior to hybridization with primary antibodies (fibronectin, LAMC1, PAI-1, and α-actinin) during western blot. MW (in kDa) are indicated. The α-actinin was used as a loading control to normalize the protein expression data.

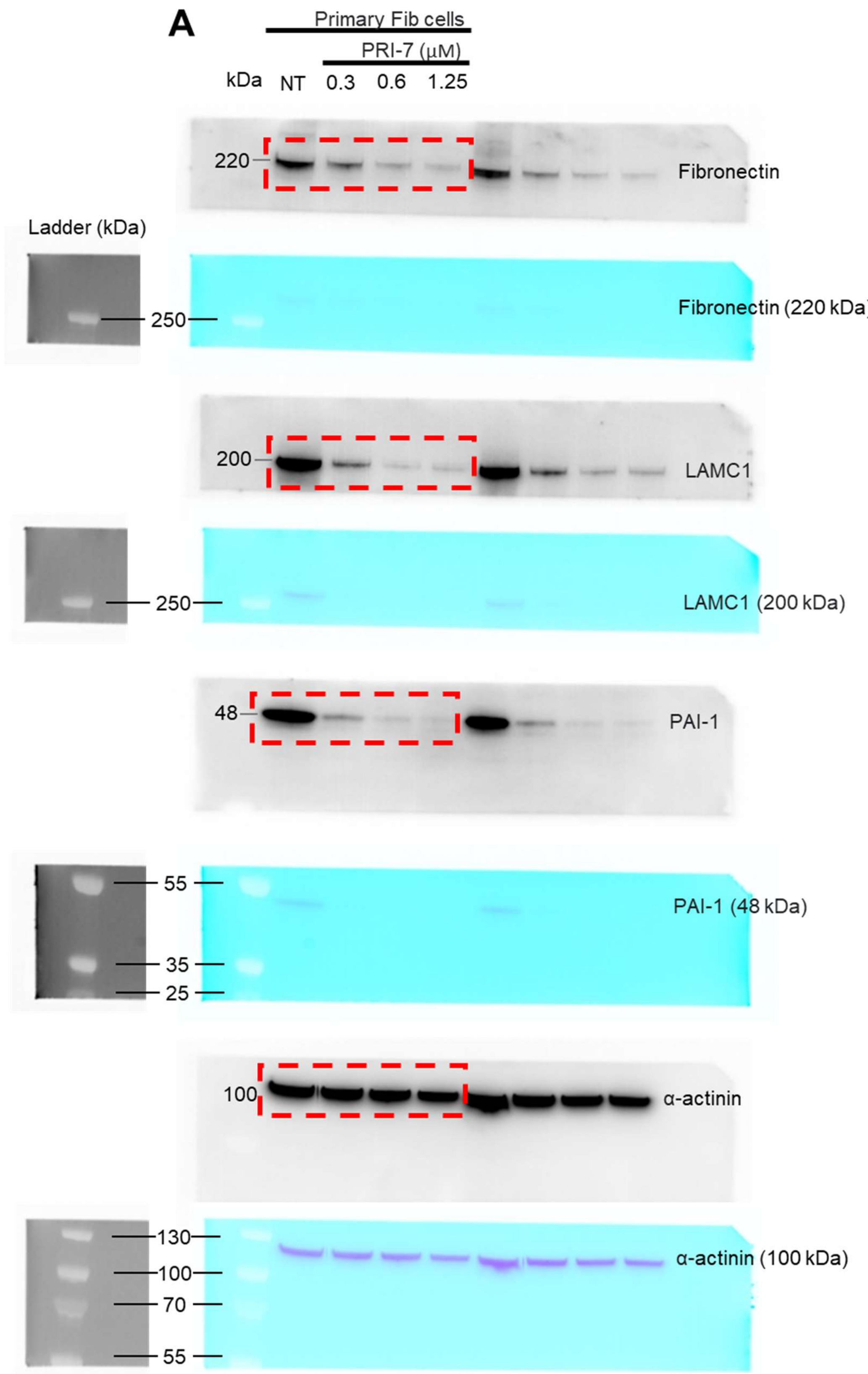

A
Primary Fib cells
PRI-7 (μM)
kDa
NT
0.3
0.6
1.25
220
Fibronectin
Ladder (kDa)
250
Fibronectin (220 kDa)
200
LAMC1
250
LAMC1 (200 kDa)
48
PAI-1
55
35
25
PAI-1 (48 kDa)
100
α-actinin
130
100
70
55
α-actinin (100 kDa)
